# Mediating role of depression medication on association between Body Mass Index and cigarette smoking among US adults: Insights from the NHIS

**DOI:** 10.1371/journal.pone.0351210

**Published:** 2026-07-08

**Authors:** Olanrewaju Onigbogi, Alperen Korkmaz, Kebba Kah, Emilee J. Delbridge, Kolawole Okuyemi

**Affiliations:** 1 Department of Family Medicine, Indiana University School of Medicine, Indianapolis, Indiana, United States of America; 2 The Gladys W. and David H. Patton College of Education, The Ohio University, Athens, Ohio, United States of America; The University of British Columbia, CANADA

## Abstract

Depression in people who are obese, and smoke cigarettes is often complicated by the possibility of using smoking as a tool for coping with stress. This study sought to determine the mediating role of depression medication on smoking among obese, overweight, normal weight and underweight adults. Data from the 2023 National Health Interview Survey, an annual public health survey of adults from 18–65 years of age in the United States of America, was analyzed using a Generalized Structural Equation Model. Underweight participants on depression medication were more likely to smoke compared to obese participants (reference group: obese; aOR = 0.49, 95% CI: [0.38, 0.63]). There was an indirect association between obesity and depression medication on smoking and obese participants on depression medication had 1.32 times higher odds of smoking when using depression medication compared to underweight individuals (aOR = 1.32, 95% CI: [1.09, 1.56]). Use of depression medication had the highest mediating role on smoking among underweight and the lowest role among obese participants (aOR = 1.64, 95% CI: [1.05, 2.22]). The findings suggest that body mass index should be considered in planning smoking cessation interventions in health care settings.

## Introduction

Studies conducted on the effect of cigarette smoking on Body Mass Index (BMI) reveal a complex relationship as overweight individuals are believed to be more likely to take up smoking, smoke more heavily and continue to smoke rather than quit, in comparison with lower weight individuals [1–3]. In addition, weight gain is commonly cited as a concern for female smokers who are considering quitting [4] although this is not usually true for male smokers [5]. The observation among adolescent males and females is different from adults as body dissatisfaction has been reported to be responsible for smoking initiation in the younger group [6, 7]. In addition, it is plausible that BMI could alter smoking rates as higher BMI results in lower blood nicotine for the same volume of cigarettes with increased smoking as a form of compensation [8]. This physiological link could also be explained by absorption of nicotine by fatty tissue making less nicotine available in the blood [9–11].

In addition, researchers have observed the link between smoking, BMI and non-communicable diseases such as Diabetes Mellitus and hypertension [12]. A better understanding of the pathway involved in this link could advance the design of intervention mechanisms to prevent Non-communicable Diseases (NCD). In addition, this understanding could improve the prognosis of NCD since both smoking and higher BMI levels are important factors in determining its course and preventing associated morbidity and mortality [13,14].

Moreover, there is evidence of an association between smoking, obesity and depression although the findings have been inconsistent [15,16]. Depression mediates the link between BMI and smoking through shared neurobiological reward pathways, maladaptive coping behaviors, and chronic inflammation. On one hand, some people use cigarettes to cope with low mood [15]. Other researchers suggest that the relationship between depression and obesity may be due to the engagement of depressed persons in unhealthy eating and sedentary behavior to cope with the condition [16]. This link could however be weak among smokers, who may use tobacco (instead of food) to cope with mood symptoms. In addition, chronic inflammation is believed to be a vital link between obesity and depression. Firstly, obesity is perceived by the brain as a stressor resulting in elevated pro-inflammatory cytokines. In addition, fat tissue in obese people is believed to contain macrophages which release inflammatory hormones such as TNF-alpha and interleukin-6 with prolonged low-level activation of the immune system and symptoms of chronic inflammation [17–21]. It is worthy to note that population-wide studies on the exclusive link between obesity and depression also present inconsistent results as heterogeneity of the samples is believed to mask variables that moderate the relationship. These variables include educational status, genetics, age, ethnicity and body image dissatisfaction especially among women [15,22–24].

Despite available evidence on the link between depression and obesity as stated above, the mediating role of depression on cigarette smoking across all BMI categories remains unclear. An understanding of this role is important in planning cigarette smoking cessation interventions in health care settings where clients may be experiencing depression.

The objective of this study, therefore, was to determine the mediating role of use of depression medication on cigarette smoking among obese, overweight, normal, and underweight adults.

## Materials and methods

### Data

This study used data from the 2023 National Health Interview Survey (NHIS), a cross-sectional survey conducted by the National Center for Health Statistics. The analysis was restricted to adult respondents aged 18–65 years from the civilian, non-institutionalized United States (U.S) population. The NHIS collects health-related information through in-person interviews conducted in households. The survey utilizes a multi-stage, clustered sampling technique using weighting specifically developed to obtain an accurate representation of the population of the U.S, with sampling weights applied to adjust for nonresponse and ensure accurate representation across demographic groups.

### Ethical compliance

This study used publicly available, de-identified data from NHIS. Ethical approval was not required per institutional guidelines. The data was accessed for research purposes on July 7, 2024. The authors did not have access to information that could identify individual participants during or after data collection.

### Measures

The outcome variable of this study was cigarette smoking, which was categorized as a binary variable (Yes/No). It was derived from cigarette smoking status: current smokers (daily or occasional) and coded as “1,” while non-smokers (former or never) were coded as “0.” Never and former smokers were grouped together into a single ‘non-smoker’ category to focus on current smoking behavior, which posed the most immediate public health risk. The main independent variable was the BMI. The BMI was a variable on the NHIS dataset with formula based on〖(Weight(kg)) ⁄(Height(m)^2^ rounded to two decimal places. BMI categories were defined as underweight (BMI < 18.5), healthy weight (BMI 18.5 to < 25), overweight (BMI ≥ 25 to < 30), and obese (BMI ≥ 30). BMI was treated as a multinomial variable for analysis. The mediator variable was depression medication, derived from whether an adult participant indicated that they took medication for depression, categorized as a binary variable (Yes/No). We used depression medication as a proxy for depression because of the unavailability of questions about diagnosis of depression in the NHIS dataset. In this analysis, the covariates were sex, race/ethnicity, education, and marital status. Sex was categorized in biological terms as either female or male. Race/ethnicity was categorized as Hispanic, Non-Hispanic White, Non-Hispanic Black, Non-Hispanic Asian, non-Hispanic American Indian/Alaska Native (AIAN), and Other/Multiple Races. Educational attainment was categorized into four levels: less than high school, high school graduate, some college, and bachelor’s degree or more and marital status was captured as married, living with a partner, neither, or unknown/refused. In all regression models, the following reference categories were used: normal weight (for BMI), non-smoker (for smoking status), no use of depression medication (for depression medication), male (for sex), non-Hispanic White (for race/ethnicity), bachelor’s degree or more (for education), and married (for marital status).

### Statistical analysis

Sociodemographic and health characteristics were reported stratified by BMI category, and standardized mean differences (SMD) which were calculated using Cohen’s w (√χ²/N) to quantify the magnitude of differences across BMI groups.

**Fig 1:**
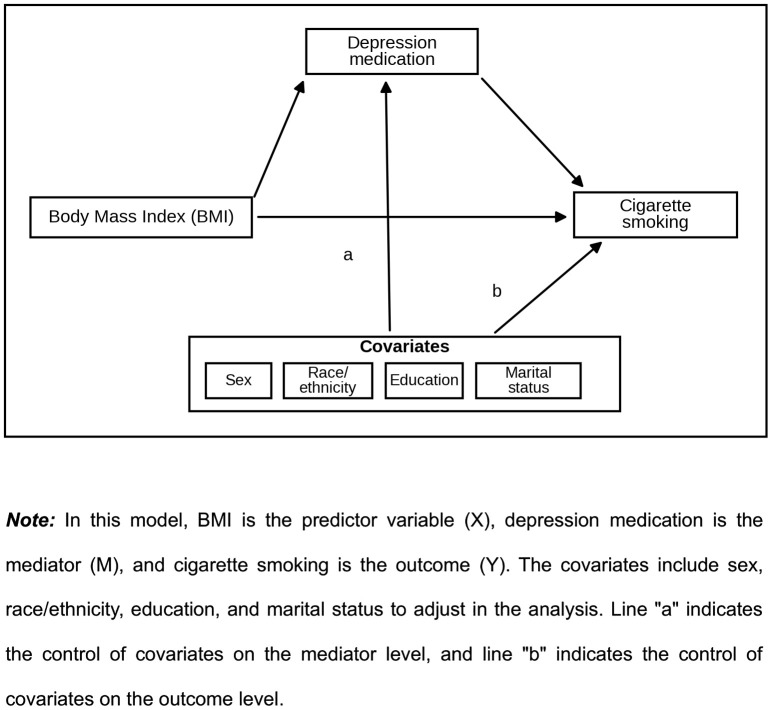
Generalized structural equation logit model examining the mediation of depression medication on the relationship between Body Mass Index and cigarette smoking.

A Generalized Structural Equation Model (GSEM) with a logit link was used to examine the mediation role of depression medication on the relationship between BMI categories and cigarette smoking (Fig 1). GSEM is a statistical model that extends the traditional Structural Equation Model (SEM) and provides flexibility in handling non-normal distributions and non-linear relationships. This model, unlike traditional SEM, has a greater flexibility to incorporate different model designs such as logistic regression with a mediator and different types of variables such as count, binary, multivalued, or continuous, for both outcomes, mediator and predictors [25].

Odds ratios were used to measure the strength of relationships along each path in the model. Direct, indirect, and total relationships were presented as typical in mediation analysis, without implying causality. Survey sampling weights were used in all analyses to account for the complicated, multistage probability sampling methodology and produce nationally representative estimations. Analysis was conducted using Stata version 18.0 (StataCorp LLC, College Station, TX).

i. *Outcome Model (Cigarette Smoking Status):*

$$\begin{array}{c} \text{log}it\left[P\left(Smoking_{i}=j\right)\right]=\beta_{\text{0}j}+\beta_{\text{1}j}*BMI_{i}+\beta_{\text{2}j}*Depression_{i}+\beta_{\text{3}j}*Covariates_{i} \end{array}$$ (1)

ii. *Mediator Model (Depression):*

$$\begin{array}{c} \text{log}it\left[P\left(Smokingi=j\right)\right]=\gamma \text{0}+\gamma \text{1}*BMIi*\gamma \text{2}*Covariates\;i \end{array}$$ (2)

In the outcome model (1), the probability of cigarette smoking status (categorized as j) was modeled using a logit model, where the log odds are a function of BMI, depression medication, and other covariates. The coefficients $\beta_{\text{1}j}$ and $\beta_{\text{2}j}$ capture the effects of each level of BMI and use of depression medication on smoking status, respectively. In the mediator model (2), use of depression medication is treated as a binary outcome, with its log odds modeled as a function of BMI and other covariates. The coefficient $\gamma_{\text{1}}$ represents the effect of each level of BMI on use of depression medication. Sociodemographic variables, including sex, race/ethnicity, education, and marital status, were included as potential confounders due to their associations with BMI, depression, and smoking. Consistent with the identification assumptions of the counterfactual mediation framework, the same set of covariates was included in both the mediator and outcome models to account for confounding of the exposure-outcome, exposure-mediator, and mediator-outcome relationships [26–28].

The mediation analysis was conducted twice, each time using a different reference group for BMI. To facilitate meaningful comparisons between BMI categories, we conducted two separate GSEM, each using a different reference group for BMI. Model 1 used underweight individuals as the reference group to assess how normal weight, overweight, and obese individuals differ in smoking behavior and its mediation role by depression medication relative to this lower-weight category. Model 2 used obese individuals as the reference group to evaluate the same mediation pathways, focusing on contrasts with participants of lower BMI.

This dual-reference modeling strategy allows for a bidirectional understanding of the association between BMI and smoking, highlighting both ends of the BMI spectrum, which have distinct clinical and public health implications. The choice of reference category in BMI studies has been shown to meaningfully influence effect estimates and their interpretation, further supporting the rationale for employing complementary reference groups to provide a more complete characterization of the BMI-smoking relationship across the full weight spectrum [29,30]. We employed the delta method to test the significance of the indirect association, allowing for accurate estimation of standard errors in mediation analysis. We employed the delta method to test the significance of the indirect association, allowing for accurate estimation of standard errors in mediation analysis [12,26–29].

As a sensitivity analysis, the counterfactual framework-based weighting approach was applied to assess the robustness of the primary findings [26]. This method decomposes the total effect of BMI on smoking into the natural direct effect (NDE), natural indirect effect (NIE), and the proportion mediated (PNIE), using logit models for both the outcome and mediator, with underweight as the reference BMI category. Sex, race/ethnicity, education, and marital status were included as covariates in both the outcome and mediator models.

## Results

Table 1 presents the descriptive characteristics of the 2023 NHIS sample (n = 28,892). The sample was almost evenly split by sex, with 53.77% male and 46.23% female. Most respondents were Non-Hispanic White (66.32%), followed by Hispanic (14.87%) and Non-Hispanic Black (10.72%). Nearly half (47.15%) had a bachelor’s degree or higher, and 44.98% were married. Most respondents were overweight (34.42%) or obese (33.37%), with 30.62% classified as having a healthy weight, and only 1.58% categorized as underweight.

**Table 1:** Sociodemographic and health characteristics of the 2023 NHIS sample stratified by Body Mass Index category (N = 29,516).

Variable	Underweight (n = 458)	Normal weight (n = 8,871)	Overweight (n = 9,938)	Obese (n = 9,625)	Total (n = 28,892)	SMD
Weighted %	1.6%	30.6%	34.0%	33.8%	100%	—
**Sex, %**						0.115
Female	66.0%	57.6%	44.1%	50.2%	50.7%	
Male	34.0%	42.4%	55.9%	49.8%	49.3%	
**Race/Ethnicity, %**						0.162
Hispanic	10.9%	14.4%	18.9%	19.0%	17.4%	
Non-Hispanic White	64.3%	63.4%	61.8%	60.7%	61.9%	
Non-Hispanic Black	10.6%	9.2%	11.1%	15.0%	11.8%	
Non-Hispanic Asian	12.0%	10.5%	6.2%	2.4%	6.3%	
Non-Hispanic AIAN	1.4%	1.0%	1.1%	1.7%	1.3%	
Other/Multiple	0.9%	1.6%	1.0%	1.2%	1.2%	
**Education, %**						0.129
Less than High School	8.0%	5.3%	5.9%	7.0%	6.1%	
High School Graduate	18.2%	15.3%	17.4%	21.0%	18.0%	
Some College	31.6%	25.7%	27.5%	33.8%	29.2%	
Bachelor’s or More	42.2%	53.6%	49.2%	38.3%	46.7%	
**Marital Status, %**						0.084
Married	38.0%	44.8%	53.9%	49.0%	49.2%	
Living with Partner	7.2%	8.5%	8.3%	8.7%	8.5%	
Neither	50.3%	42.0%	33.7%	38.5%	38.1%	
Unknown/Refused	4.4%	4.6%	4.1%	3.8%	4.2%	
**Depression Medication, %**						0.077
Yes	10.6%	9.3%	9.9%	14.9%	11.4%	
No	89.4%	90.7%	90.1%	85.1%	88.6%	
**Current Smoking, %**						0.043
Yes	19.8%	11.6%	9.7%	11.0%	10.9%	
No	80.2%	88.5%	90.3%	89.0%	89.1%	

All percentages are weighted to account for the complex survey design of the NHIS. Excluded cases = 624. SMD = Standardized Mean Difference (Cohen’s w = √(χ²/N)); SMD ≥ 0.10 indicates meaningful imbalance between groups. AIAN = American Indian or Alaska Native. Body Mass Index (BMI) categories: Underweight (<18.5), Normal weight (18.5– < 25), Overweight (25– < 30), Obese (≥30).

Table 2 presents the GSEM results for the role of depression medication on the relationship between each BMI category. In Model 1, the direct association of being underweight compared to normal weight, overweight, and obese on smoking cigarettes is statistically significant. Obese individuals had the lowest odds of smoking, followed by overweight and normal weight individuals. Underweight individuals had significantly higher odds of smoking compared to all other BMI groups (Model 1: underweight aOR = 0.49, 95% CI: [0.38, 0.63]; normal weight aOR = 0.35, 95% CI: [0.27, 0.44]; obese aOR = 0.34, 95% CI: [0.27, 0.44]). In Model 2, the direct association of being obese compared to underweight and normal weight on smoking cigarettes was also statistically significant, while the comparison with overweight individuals was not significant. Obese individuals had significantly higher odds of smoking than underweight and normal-weight individuals (Model 2: underweight aOR = 2.26, 95% CI: [1.67, 3.05]; normal weight aOR = 1.32, 95% CI: [1.18, 1.48]).

**Table 2:** Generalized structural equation model logit model analysis of the mediating role of depression medication on the relationship between Body Mass Index categories and smoking status.

Paths	Direct Association aOR [95% CI]	Indirect Association aOR [95% CI]	Total Association aOR [95% CI]
**Model 1 (Reference = Underweight)**			
Underweight- > Depression Medication **- >** Smoking	**Ref**	**Ref**	**Ref**
Normal weight- > Depression Medication- > Smoking	0.49 [0.38, 0.63] *	0.93 [0.77, 1.09]	0.46 [0.32, 0.59] *
Overweight - > Depression medication- > Smoking	0.35 [0.27, 0.44] *	1.06 [0.88, 1.23]	0.37 [0.26, 0.48] *
Obese- > Depression Medication- > Smoking	0.34 [0.27, 0.44] *	1.32 [1.09, 1.56] *	0.45 [0.31, 0.59] *
**Model 2 (Reference = Obese)**			
Underweight- > Depression Medication- > Smoking	2.26 [1.67, 3.05] *	0.72 [0.57, 0.88] *	1.64 [1.05, 2.22] *
Normal weight- > Depression Medication - > Smoking	1.32 [1.18, 1.48] *	0.70 [0.63, 0.77] *	0.93 [0.79, 1.06]
Overweight - > Depression Medication - > Smoking	1.01 [0.90, 1.13]	0.80 [0.74, 0.85] *	0.80 [0.69, 0.91] *
Obese - > Depression Medication - > Smoking	**Ref**	**Ref**	**Ref**

Model 1: underweight as reference category; Model 2: obese as reference category. Different reference categories were used to facilitate bidirectional comparisons across the full BMI spectrum.

* Indicates statistical significance at *p* < 0.001.

aOR represents adjusted odds ratio.

95% CI denotes 95% Confidence Interval.

“Ref” indicates the reference category used for comparison in each model.

In Model 1, the indirect association of depression medication on smoking was statistically significant only for obese individuals, who had 1.32 times higher odds of smoking when using depression medication compared to underweight individuals (aOR = 1.32, 95% CI: [1.09, 1.56]). In Model 2, the indirect association of depression medication on smoking was statistically significant for underweight, normal-weight, and overweight individuals when compared to obese individuals. Normal-weight individuals on depression medication had 0.70 times lower odds of smoking compared to obese individuals on depression medication (aOR = 0.70, 95% CI: [0.63, 0.77]), and overweight individuals on depression medication had 0.80 times lower odds of smoking compared to obese individuals on depression medication (aOR = 0.80, 95% CI: [0.74, 0.85]).

In Model 1, the total association showed that normal-weight, overweight, and obese individuals had significantly lower odds of smoking compared to underweight individuals, with adjusted odds ratios (aOR) of 0.46 (95% CI: [0.32, 0.59]), 0.37 (95% CI: [0.26, 0.48]), and 0.45 (95% CI: [0.31, 0.59]), respectively. In Model 2, underweight individuals had significantly higher odds of smoking compared to obese individuals (aOR = 1.64, 95% CI: [1.05, 2.22]), while overweight individuals had significantly lower odds (aOR = 0.80, 95% CI: [0.69, 0.91]), and the total association for normal-weight individuals was not statistically significant (aOR = 0.93, 95% CI: [0.79, 1.06]).

As a sensitivity analysis, we conducted a counterfactual mediation analysis using a weighting-based framework (S1 Table), and the results were consistent with the primary GSEM findings. The natural direct effect (NDE) showed that normal weight (OR = 0.51, 95% CI: [0.41, 0.65]), overweight (OR = 0.37, 95% CI: [0.29, 0.46]), and obese individuals (OR = 0.36, 95% CI: [0.29, 0.46]) had significantly lower odds of current smoking compared to underweight individuals (all p < 0.001), while the natural indirect effect (NIE) through depression medication was statistically significant only for obese individuals (OR = 1.03, 95% CI: [1.01, 1.06], p = 0.002), consistent with the primary analysis which also identified the obese group as the only BMI category with a significant indirect effect. The total effect estimates were similarly consistent with the primary analysis across all BMI categories, and the proportion mediated (PNIE) was small and not statistically significant. This suggests that depression medication accounts for a negligible portion of the total effect of BMI on smoking.

## Discussion

The relationship between depression and obesity varies by age, gender, and ethnicity. Previous works have highlighted the effect of these moderators on the relationship between depression and obesity by utilizing cross-sectional data [28–30]. Our study is unique because it examined the association between obesity and the intersection between depression and cigarette smoking in a nationally representative sample of adults in the U.S. The near equal age and sex distribution of participants in our study is consistent with what has been observed in prior large national datasets [15,30,31]. Other studies have attempted to use longitudinal data to establish the temporal sequencing between obesity and depression, that is, to determine whether obesity leads to a change in psychological well-being or whether symptoms of depression lead to weight changes [17–19]. Another unique contribution of the study is that it assesses the effect of a mediator (i.e., use of depression medication) on cigarette smoking in people who belong to the various BMI categories classified in our study (underweight, healthy weight, overweight and obese). Previous studies have mainly focused on obesity as the only important BMI category in the relationship [17–19,21].

This study used depression medication as a proxy for depression, which aligns with other studies which found that medication use was correlated with depression severity in adults [32,33]. In addition, our study made use of GSEM with a logit link to examine the mediating role of depression medication on the relationship between all other variables and provides flexibility in handling non-normal distributions and non-linear relationship [34]. We were therefore able to create several models which we used to measure the strength of relationships along each path with direct, indirect, and total association, without assuming causality.

The results from our GSEM models do not align with the results of the study conducted using a similar methodology in the Netherlands [35]. In our Model 1, the adjusted odds ratios were used to assess the direct association of being underweight compared to normal weight, overweight, and obese on cigarette smoking; whereas, our Model 2 examined the indirect association of depression medication on smoking across all BMI categories, as well as the combined total association of use of depression medication on smoking across normal weight, overweight, and obesity categories. Our results do not show any sex-specific susceptibility in the relationship between depression and smoking across all BMI categories. However, the Dutch study found that adult women may be more vulnerable to nicotine-induced symptoms of depression across all BMI categories, potentially due to a combination of biological, psychological, and social factors [35]. This difference may be a result of the cultural contexts of the countries in which the studies took place. The smoking rates in the Netherlands is higher than what obtains in the US and adult smokers are generally less likely to believe smoking is harmful which creates a scenario of higher prevalence of nicotine-driven depression compared to the USA, where smoking is stigmatized [36].

This study identified that underweight individuals had significantly higher odds of smoking than those in other BMI groups. Likewise, obese individuals had significantly higher odds of smoking than underweight and normal-weight individuals. These results are consistent with several prior studies using similar large, population-based datasets in the U.S. [32,34,37]. A U-shaped relationship was observed between BMI and smoking, where those with either low or high BMI had higher smoking rates, while those with moderate BMI had lower rates [38]. The U-shaped relationship between BMI and smoking which we observed may be because low BMI people smoke for weight management whereas high BMI individuals often smoke due to stress/social factors. In contrast however, moderate BMI individuals tend to have healthier habits. Furthermore, low BMI smokers also often use nicotine for appetite suppression and metabolic control, while high BMI (overweight/obese) smokers are frequently linked to stress-related smoking, lower physical activity, or genetic factors affecting both BMI and cigarette smoking.

In addition, in our study, overweight individuals [BMI ≥ 25 to < 30] on depression medication had lower odds of smoking compared to obese individuals [BMI ≥ 30]. This finding aligns with previous studies which have attempted to explain that this difference in smoking predilection may be attributed to coping mechanisms, weight concerns, and the potential role of smoking as a way to regulate affect and negative emotions [39,40]. The explanation for this observation could also be related to biological and psychological mechanisms underlying this relationship, which include the impact of nicotine on the brain and the psychological mechanisms through which depressed individuals may use smoking as a coping strategy.

## Conclusions

Our study found that use of depression medication was associated with current smoking only among participants who were obese. The findings show that depression medication had the highest mediation association on smoking among underweight and lowest mediation association among obese participants. This link suggests that BMI should be considered in planning smoking cessation interventions in health care settings where clients may be experiencing depression.

## Limitations

Our study has some limitations because we utilized an existing dataset, about which we were not involved in the development of the items nor were we involved in distribution of the survey. First, its cross-sectional design prevents establishing the temporal ordering between BMI, depression medication use, and smoking behavior. Because exposure, mediator, and outcome were measured at the same time, we cannot determine whether BMI preceded depression medication use or whether depression medication use preceded smoking. This limitation is particularly important for mediation analysis, which relies on the assumption that the exposure precedes the mediator, and the mediator precedes the outcome [29]. Although the counterfactual mediation analysis provides a more rigorous decomposition of effects, it does not resolve this temporal ambiguity. Therefore, the findings should be interpreted as associations consistent with mediation rather than definitive causal pathways. Future longitudinal studies are needed to confirm the directionality and causal nature of the observed relationships. Secondly, we identify the limitations associated with the use of BMI as a measure of good or bad health because it is a poor indicator of the percentage of body fat and does not capture information on fat distribution [19]. In addition, the use of self-reported smoking data may have been a limitation in this study as self-reported smoking has been shown to underestimate actual smoking in other studies [41]. Finally, we are aware of the limitation presented by participants’ self-report of the use of prescription medication for depression as our basis for classification of participants having depression. We acknowledge that pharmacotherapy is only one of the approaches to treating depression. We also acknowledge the fact that depression is experienced on a spectrum and is clinically assessed using a scale (often from mild to severe). Since individuals may have depression without depression medication being prescribed, the criteria used in this study may not include all individuals with depression. Our study likely excluded people with undiagnosed or untreated depression, those receiving non-pharmacological interventions such as psychotherapy, or individuals with limited access to mental health care. We therefore suggest that future studies should use validated scales such as the Patient Health Questionnaire-9 (PHQ-9), the clinician-rated Hamilton Depression Rating Scale (HAM-D), or the Beck Depression Inventory (BDI) which are better proxies for depression.

## Supporting information

S1 Table Sensitivity analysis: Counterfactual framework-based causal mediation analysis of the association between Body Mass Index (BMI) and current smoking status, mediated through depression. (DOCX)

## References

[1] Audrain-McGovern J, Benowitz NL. Cigarette smoking, nicotine, and body weight. Clin Pharmacol Ther. 2011;90(1):164–8. doi: 10.1038/clpt.2011.105 21633341 PMC3195407

[2] Ma J, Betts NM, Hampl JS. Clustering of lifestyle behaviors: the relationship between cigarette smoking, alcohol consumption, and dietary intake. Am J Health Promot. 2000;15(2):107–17. doi: 10.4278/0890-1171-15.2.107 11194694

[3] Healton CG, Vallone D, McCausland KL, Xiao H, Green MP. Smoking, obesity, and their co-occurrence in the United States: cross sectional analysis. BMJ. 2006;333(7557):25–6. doi: 10.1136/bmj.38840.608704.80 16698804 PMC1488756

[4] Farley AC, Hajek P, Lycett D, Aveyard P. Interventions for preventing weight gain after smoking cessation. Cochrane Database Syst Rev. 2012;1:CD006219. doi: 10.1002/14651858.CD006219.pub3 22258966

[5] Clark MM, Decker PA, Offord KP, Patten CA, Vickers KS, Croghan IT, et al. Weight concerns among male smokers. Addict Behav. 2004;29(8):1637–41. doi: 10.1016/j.addbeh.2004.02.034 15451131

[6] Dare S, Mackay DF, Pell JP. Relationship between smoking and obesity: a cross-sectional study of 499,504 middle-aged adults in the UK general population. PLoS One. 2015;10(4):e0123579. doi: 10.1371/journal.pone.0123579 25886648 PMC4401671

[7] Morris RW, Taylor AE, Fluharty ME, Bjørngaard JH, Åsvold BO, Elvestad Gabrielsen M, et al. Heavier smoking may lead to a relative increase in waist circumference: evidence for a causal relationship from a Mendelian randomisation meta-analysis. The CARTA consortium. BMJ Open. 2015;5(8):e008808. doi: 10.1136/bmjopen-2015-008808 26264275 PMC4538266

[8] Winter A-L, de Guia NA, Ferrence R, Cohen JE. The relationship between body weight perceptions, weight control behaviours and smoking status among adolescents. Can J Public Health. 2002;93(5):362–5. doi: 10.1007/BF03404570 12353458 PMC6979969

[9] Tomeo CA, Field AE, Berkey CS, Colditz GA, Frazier AL. Weight concerns, weight control behaviors, and smoking initiation. Pediatrics. 1999;104(4 Pt 1):918–24. doi: 10.1542/peds.104.4.918 10506235

[10] Jain RB, Bernert JT. Effect of body mass index and total blood volume on serum cotinine levels among cigarette smokers: NHANES 1999-2008. Clin Chim Acta. 2010;411(15–16):1063–8. doi: 10.1016/j.cca.2010.03.040 20361952

[11] Prather RD, Tu TG, Rolf CN, Gorsline J. Nicotine pharmacokinetics of Nicoderm (nicotine transdermal system) in women and obese men compared with normal-sized men. J Clin Pharmacol. 1993;33(7):644–9. doi: 10.1002/j.1552-4604.1993.tb04718.x 8366189

[12] Nuttall FQ. Body mass index: obesity, BMI, and health: a critical review. Nutr Today. 2015;50(3):117–28. doi: 10.1097/NT.0000000000000092 27340299 PMC4890841

[13] Fields LJ, Roberts W, Schwing I, McCoy M, Verplaetse TL, Peltier MR, et al. Examining the relationship of concurrent obesity and tobacco use disorder on the development of substance use disorders and psychiatric conditions: Findings from the NESARC-III. Drug Alcohol Depend Rep. 2023;7:100162. doi: 10.1016/j.dadr.2023.100162 37159814 PMC10163607

[14] Chatkin R, Mottin CC, Chatkin JM. Smoking among morbidly obese patients. BMC Pulm Med. 2010;10:61. doi: 10.1186/1471-2466-10-61 21106095 PMC3004817

[15] Strine TW, Mokdad AH, Dube SR, Balluz LS, Gonzalez O, Berry JT, et al. The association of depression and anxiety with obesity and unhealthy behaviors among community-dwelling US adults. Gen Hosp Psychiatry. 2008;30(2):127–37. doi: 10.1016/j.genhosppsych.2007.12.008 18291294

[16] van den Broek N, Treur JL, Larsen JK, Verhagen M, Verweij KJH, Vink JM. Causal associations between body mass index and mental health: a Mendelian randomisation study. J Epidemiol Community Health. 2018;72(8):708–10. doi: 10.1136/jech-2017-210000 29666151

[17] Faith MS, Matz PE, Jorge MA. Obesity-depression associations in the population. J Psychosom Res. 2002;53(4):935–42. doi: 10.1016/s0022-3999(02)00308-2 12377306

[18] Stunkard AJ, Faith MS, Allison KC. Depression and obesity. Biol Psychiatry. 2003;54(3):330–7. doi: 10.1016/s0006-3223(03)00608-5 12893108

[19] Bastard J-P, Maachi M, Lagathu C, Kim MJ, Caron M, Vidal H, et al. Recent advances in the relationship between obesity, inflammation, and insulin resistance. Eur Cytokine Netw. 2006;17(1):4–12. 16613757

[20] Gavin AR, Simon GE, Ludman EJ. The association between obesity, depression, and educational attainment in women: the mediating role of body image dissatisfaction. J Psychosom Res. 2010;69(6):573–81. doi: 10.1016/j.jpsychores.2010.05.001 21109045 PMC3062479

[21] Simon GE, Von Korff M, Saunders K, Miglioretti DL, Crane PK, van Belle G, et al. Association between obesity and psychiatric disorders in the US adult population. Arch Gen Psychiatry. 2006;63(7):824–30. doi: 10.1001/archpsyc.63.7.824 16818872 PMC1913935

[22] Heo M, Pietrobelli A, Fontaine KR, Sirey JA, Faith MS. Depressive mood and obesity in US adults: comparison and moderation by sex, age, and race. Int J Obes (Lond). 2006;30(3):513–9. doi: 10.1038/sj.ijo.0803122 16302017

[23] Fuemmeler BF, Agurs-Collins T, McClernon FJ, Kollins SH, Garrett ME, Ashley-Koch AE. Interactions between genotype and depressive symptoms on obesity. Behav Genet. 2009;39(3):296–305. doi: 10.1007/s10519-009-9266-z 19337825 PMC2884968

[24] Zhao G, Ford ES, Dhingra S, Li C, Strine TW, Mokdad AH. Depression and anxiety among US adults: associations with body mass index. Int J Obes (Lond). 2009;33(2):257–66. doi: 10.1038/ijo.2008.268 19125163

[25] Kleinbaum DG, Klein M. Logistic Regression: A Self-Learning Text. 3rd ed. New York: Springer. 2010. doi: 10.1007/978-1-4419-1742-3

[26] Vanderweele TJ, Vansteelandt S. Conceptual issues concerning mediation, interventions and composition. Statistics and Its Interface. 2009;2(4):457–68. doi: 10.4310/sii.2009.v2.n4.a7

[27] Valeri L, Vanderweele TJ. Mediation analysis allowing for exposure-mediator interactions and causal interpretation: theoretical assumptions and implementation with SAS and SPSS macros. Psychol Methods. 2013;18(2):137–50. doi: 10.1037/a0031034 23379553 PMC3659198

[28] Azagba S, Ebling T, Korkmaz A. Social media and e-cigarette use: The mediating role of mental health conditions. J Affect Disord. 2024;344:528–34. doi: 10.1016/j.jad.2023.10.053 37852589

[29] Flegal KM, Kit BK, Graubard BI. Body mass index categories in observational studies of weight and risk of death. Am J Epidemiol. 2014;180(3):288–96. doi: 10.1093/aje/kwu111 24893710 PMC4732880

[30] Pavela G, Yi N, Mestre L, Lartey S, Xun P, Allison DB. The associations between relative and absolute body mass index with mortality rate based on predictions from stigma theory. SSM Popul Health. 2022;19:101200. doi: 10.1016/j.ssmph.2022.101200 36033349 PMC9399523

[31] Hosmer DW, Lemeshow S, Sturdivant RX. Applied Logistic Regression: 3rd ed. Wiley. 2013. doi: 10.1002/9781118548387

[32] Zhao G, Ford ES, Li C, Strine TW, Dhingra S, Berry JT, et al. Serious psychological distress and its associations with body mass index: findings from the 2007 Behavioral Risk Factor Surveillance System. Int J Public Health. 2009;54 Suppl 1:30–6. doi: 10.1007/s00038-009-0004-3 19424662

[33] Wu Z, Yue Q, Zhao Z, Wen J, Tang L, Zhong Z. A cross-sectional study of smoking and depression among US adults: NHANES (2005-2018). Front Public Health. 2023;11:1081706. doi: 10.3389/fpubh.2023.1081706 36794066 PMC9922891

[34] Flegal KM, Kit BK, Orpana H, Graubard BI. Association of all-cause mortality with overweight and obesity using standard body mass index categories: a systematic review and meta-analysis. JAMA. 2013;309(1):71–82. doi: 10.1001/jama.2012.113905 23280227 PMC4855514

[35] van Hooijdonk KJM, van den Broek N, Tan CY, Vink JM, Larsen J. Current Nicotine use and the Development of Depressive Symptoms Across Adolescence and Adulthood: A Multi-Dataset Study Exploring Moderation Effects of Body Mass Index and Sex. Int J Ment Health Addiction. 2025;24(2):1755–73. doi: 10.1007/s11469-025-01484-4

[36] Evans-Polce RJ, Castaldelli-Maia JM, Schomerus G, Evans-Lacko SE. The downside of tobacco control? Smoking and self-stigma: A systematic review. Soc Sci Med. 2015;145:26–34. doi: 10.1016/j.socscimed.2015.09.026 26439764 PMC4630105

[37] Long J, Freese J. Regression Models for Categorical Dependent Variables Using Stata. 3rd ed. College Station, TX: Stata Press. 2014.

[38] Widome R, Linde JA, Rohde P, Ludman EJ, Jeffery RW, Simon GE. Does the association between depression and smoking vary by body mass index (BMI) category?. Prev Med. 2009;49(5):380–3. doi: 10.1016/j.ypmed.2009.07.018 19647015 PMC2784124

[39] Leventhal AM, Mickens L, Dunton GF, Sussman S, Riggs NR, Pentz MA. Tobacco use moderates the association between major depression and obesity. Health Psychol. 2010;29(5):521–8. doi: 10.1037/a0020854 20836607 PMC3204861

[40] Kaufman A, Augustson EM, Patrick H. Unraveling the Relationship between Smoking and Weight: The Role of Sedentary Behavior. J Obes. 2012;2012:735465. doi: 10.1155/2012/735465 21961058 PMC3180774

[41] Connor Gorber S, Schofield-Hurwitz S, Hardt J, Levasseur G, Tremblay M. The accuracy of self-reported smoking: a systematic review of the relationship between self-reported and cotinine-assessed smoking status. Nicotine Tob Res. 2009;11(1):12–24. doi: 10.1093/ntr/ntn010 19246437

