# Supplementary material for: Mediating role of depression medication on association between Body Mass Index and cigarette smoking among US adults: Insights from the NHIS
Source: PLoS One. 2026 Jul 8;21(7):e0351210. doi: 10.1371/journal.pone.0351210 (PMC13345264; doi:10.1371/journal.pone.0351210)
Supplement: S1 Table — (DOCX) [file pone.0351210.s001.docx]

**Table S1. Sensitivity Analysis:** Counterfactual Framework-Based Causal Mediation Analysis of the Association between Body Mass Index and Current Smoking Status, Mediated through Depression

| **BMI Category** | **OR (SE)** | **95% CI** | **p-value** |
| --- | --- | --- | --- |
| **Natural Indirect Effect (NIE)** | | | |
| Normal weight vs. underweight | 0.99 (0.009) | 0.976, 1.009 | 0.359 |
| Overweight vs. underweight | 1.01 (0.010) | 0.986, 1.025 | 0.596 |
| Obese vs. underweight | 1.03 (0.011) | 1.013, 1.056 | 0.002 |
| **Natural Direct Effect (NDE)** | | | |
| Normal weight vs. underweight | 0.51 (0.060) | 0.410, 0.645 | <0.001 |
| Overweight vs. underweight | 0.37 (0.043) | 0.294, 0.464 | <0.001 |
| Obese vs. underweight | 0.36 (0.042) | 0.289, 0.456 | <0.001 |
| **Proportion Mediated via NIE (PNIE)** | | | |
| Normal weight vs. underweight | 1.00 (0.004) | 0.991, 1.008 | 0.845 |
| Overweight vs. underweight | 1.00 (0.003) | 0.995, 1.006 | 0.850 |
| Obese vs. underweight | 1.00 (0.017) | 0.970, 1.038 | 0.841 |
| **Total Natural Direct Effect (TNDE)** | | | |
| Normal weight vs. underweight | 0.51 (0.059) | 0.407, 0.641 | <0.001 |
| Overweight vs. underweight | 0.37 (0.043) | 0.296, 0.466 | <0.001 |
| Obese vs. underweight | 0.37 (0.043) | 0.298, 0.470 | <0.001 |
| **Total Effect (TE)** | | | |
| Normal weight vs. underweight | 0.51 (0.059) | 0.407, 0.640 | <0.001 |
| Overweight vs. underweight | 0.37 (0.043) | 0.296, 0.466 | <0.001 |
| Obese vs. underweight | 0.38 (0.044) | 0.299, 0.471 | <0.001 |

Notes. OR = Odds Ratio; SE = Standard Error; CI = Confidence Interval. NIE = Natural Indirect Effect; NDE = Natural Direct Effect; PNIE = Proportion Mediated via NIE; TNDE = Total Natural Direct Effect; TE = Total Effect. Reference category: underweight (BMI < 18.5).
