# Supplementary material for: Mediating role of depression medication on association between Body Mass Index and cigarette smoking among US adults: Insights from the NHIS
Source: PLoS One. 2026 Jul 8;21(7):e0351210. doi: 10.1371/journal.pone.0351210 (PMC13345264; doi:10.1371/journal.pone.0351210)
Supplement: Attachment — Submitted filename: comment.docx [file pone.0351210.s002.docx]

**. Conceptual Clarity of Mediation Framework**

- The manuscript explores depression as a mediator between BMI and smoking, but the theoretical rationale for this pathway needs clearer justification.
- **Recommendation**: Strengthen the conceptual model in the Introduction by explaining *why* depression might mediate the BMI–smoking link, not just correlate with both. Consider citing behavioral or neurobiological mechanisms that support this mediation.

**2. Measurement of Depression**

- Depression is measured via a single binary item: use of prescription medication. This may not capture the full spectrum of depressive symptoms or severity.
- **Recommendation**: Acknowledge this limitation explicitly in the Discussion. Consider discussing how this proxy may underestimate or misclassify depression, and suggest future studies use validated multi-item scales.

**3. Interpretation of Mediation Effects**

- The manuscript reports odds ratios for indirect effects but does not clearly explain how these were calculated or interpreted.
- **Recommendation**: Clarify the mediation analysis framework (e.g., product of coefficients, delta method) and explain how indirect effects were derived and interpreted in the context of GSEM.

**4. Model Specification and Reference Groups**

- Two models are used with different BMI reference categories (underweight and obese), but the rationale for this dual approach is not fully explained.
- **Recommendation**: Justify the use of dual reference groups in the Methods and clarify how this enhances interpretability or addresses clinical relevance.

**5. Generalizability and Sample Description**

- The sample is drawn from NHIS, but the manuscript inconsistently refers to it as the “National Youth Tobacco Survey” in Table 1.
- **Recommendation**: Correct this labeling and ensure consistent terminology throughout. Also, discuss generalizability given the cross-sectional design and self-reported data.

**6. Ethical Statement and Data Access**

- The ethics section states that IRB approval was not required due to public data use, but this should be framed more formally.
- **Recommendation**: Rephrase to: “This study used publicly available, de-identified data from the NHIS. Ethical approval was not required per institutional guidelines.”

**7. Language and Formatting**

- Several sections contain grammatical errors, inconsistent tense, and formatting issues (e.g., spacing, punctuation, equation formatting).
- **Recommendation**: Conduct a thorough language and style edit before resubmission. Ensure equations are properly typeset and tables are consistently labeled.

**8**. **Formatting**

- Check the plos one journal instruction
