# Supplementary material for: Mediating role of depression medication on association between Body Mass Index and cigarette smoking among US adults: Insights from the NHIS
Source: PLoS One. 2026 Jul 8;21(7):e0351210. doi: 10.1371/journal.pone.0351210 (PMC13345264; doi:10.1371/journal.pone.0351210)
Supplement: Attachment — Submitted filename: 11th Dec Response to Reviewers.docx [file pone.0351210.s005.docx]

Response to Editor and reviewers

Dear Editor,

Thank you for the opportunity to review and submit our paper once again to PLOS One journal taking into consideration the response from the reviewers.

In addition, we have changed the title to ‘Mediating role of depression medication on association between Body Mass Index and cigarette smoking among US adults: Insights from the NHIS’ to reflect the comments by reviewers.

The authors have made the changes as suggested by the reviewers. Our responses are highlighted below.

Thank you.

Olanrewaju Onigbogi

09 December 2025

Reviewer #1: Conceptual Clarity of Mediation Framework
1. The manuscript explores depression as a mediator between BMI and smoking, but the theoretical rationale for this pathway needs clearer justification.
• Recommendation: Strengthen the conceptual model in the Introduction by explaining why depression might mediate the BMI–smoking link, not just correlate with both. Consider citing behavioral or neurobiological mechanisms that support this mediation.

Response:

Thank you for this observation. To further strengthen the conceptual model for our study, we added some detail about how depression mediates the link between BMI and smoking through shared neurobiological reward pathways, maladaptive coping behaviors, and chronic inflammation (Page 4, Lines 92-95).

2. Measurement of Depression
• Depression is measured via a single binary item: use of prescription medication. This may not capture the full spectrum of depressive symptoms or severity.
• Recommendation: Acknowledge this limitation explicitly in the Discussion. Consider discussing how this proxy may underestimate or misclassify depression, and suggest future studies use validated multi-item scales.

Response:

Thank you for the comments. We have acknowledged this limitation in the discussion highlighting why using depression medication as a proxy may underestimate or misclassify depression. We have also suggested that future studies use validated scales such as the Patient Health Questionnaire-9 (PHQ-9) for self-report screening, the clinician-rated Hamilton Depression Rating Scale (HAM-D), and the Beck Depression Inventory (BDI) – (Page 18, Lines 66-82).

3. Interpretation of Mediation Effects
• The manuscript reports odds ratios for indirect effects but does not clearly explain how these were calculated or interpreted.
• Recommendation: Clarify the mediation analysis framework (e.g., product of coefficients, delta method) and explain how indirect effects were derived and interpreted in the context of GSEM.

Response:

Thank you for this important comment. We agree that the original description of the mediation framework needed additional clarification. In the revised manuscript, we expanded the Methods section under ‘Statistical Analysis’ to explain how indirect association were computed in the GSEM framework and how odds ratios were derived and interpreted (Page 8, Lines 160-171).

4. Model Specification and Reference Groups
• Two models are used with different BMI reference categories (underweight and obese), but the rationale for this dual approach is not fully explained.
• Recommendation: Justify the use of dual reference groups in the Methods and clarify how this enhances interpretability or addresses clinical relevance.

Response:

Thank you for this comment. We clarified the rationale for using two BMI reference groups. Methodological guidance supports using alternative reference categories to improve interpretability of categorical predictors (Hosmer & Lemeshow, 2013; Kleinbaum & Klein, 2010; Long & Freese, 2014). Because underweight and obese represent clinically distinct extremes of the BMI spectrum, analyzing both as reference groups provides meaningful contrasts in both directions and avoids a one-sided comparison (Nuttall, 2015; Flegal et al., 2013). This explanation has been added to the Methods section (Page 9, Lines 185-196).

References:
 Hosmer DW, Lemeshow S, Sturdivant RX. *Applied Logistic Regression*. 3rd ed. Wiley; 2013.

Kleinbaum DG, Klein M. *Logistic Regression: A Self-Learning Text*. 3rd ed. Springer; 2010.

Long JS, Freese J. *Regression Models for Categorical Dependent Variables Using Stata*. 3rd ed. College Station, TX: Stata Press; 2014.

Nuttall FQ. Body Mass Index: Obesity, BMI, and Health: A Critical Review. *Nutr Today*. 2015;50(3):117–128.

Flegal KM, Kit BK, Orpana H, Graubard BI. Association of All-Cause Mortality With Overweight and Obesity Using Standard Body Mass Index Categories: A Systematic Review and Meta-analysis. *JAMA*. 2013;309(1):71–82.

5. Generalizability and Sample Description
• The sample is drawn from NHIS, but the manuscript inconsistently refers to it as the “National Youth Tobacco Survey” in Table 1.
• Recommendation: Correct this labeling and ensure consistent terminology throughout. Also, discuss generalizability given the cross-sectional design and self-reported data.

Response:

Thank you for noting this inconsistency. We corrected the labeling in Table 1, replacing “National Youth Tobacco Survey” with the correct data source, the National Health Interview Survey (NHIS), and reviewed the manuscript to ensure consistent terminology throughout (Page12).

We also added a brief statement on generalizability, noting that findings are limited by the cross-sectional design and reliance on self-reported measures, which may introduce reporting bias and prevent causal inference. This clarification has been added to the Discussion section (Page 16, Lines 25-40).

6. Ethical Statement and Data Access
• The ethics section states that IRB approval was not required due to public data use, but this should be framed more formally.
• Recommendation: Rephrase to: “This study used publicly available, de-identified data from the NHIS. Ethical approval was not required per institutional guidelines.”

Response:

Thank you for this observation and do agree that the suggested text is a better expression of what we did. We have quoted the suggested phrase verbatim and added to the text in the Ethical compliance section (Page 6, Lines 131-132)

7. Language and Formatting
• Several sections contain grammatical errors, inconsistent tense, and formatting issues (e.g., spacing, punctuation, equation formatting).
• Recommendation: Conduct a thorough language and style edit before resubmission. Ensure equations are properly typeset and tables are consistently labeled.

Response:

Thank you for your observation. We have edited the document to address grammatical errors, inconsistent tense, and formatting issues. We have also redone the equation and tables (Page 8, Lines 121-126, Page 12 Lines 248-249).

8. Formatting
• Check the plos one journal instruction

Response:

Thank you for your comments. We have revisited the POLS One site addressing formatting issues in line with instructions.

Reviewer #2: Dear Authors,
This manuscript investigates the mediating role of depression in the association between BMI and cigarette smoking using the 2023 National Health Interview Survey (NHIS). The topic is of high relevance to public health, and the inclusion of all BMI categories (underweight, normal, overweight, obese) provides valuable detail. The use of a GSEM strengthens the methodological framework. However, several key methodological and interpretive limitations must be addressed before the manuscript can be considered technically sound. This review supports a Major Revision recommendation.

1-The primary methodological concern pertains to the measurement of depression. In this study, depression was identified solely through participants’ self-reported use of prescribed antidepressant medication. This approach does not capture the full spectrum of depressive symptoms; rather, it reflects only individuals currently receiving pharmacological treatment.
As a result, several issues arise. First, it likely excludes people with undiagnosed or untreated depression, those receiving non-pharmacological interventions such as psychotherapy, or individuals with limited access to mental health care. This selective definition could bias the observed associations between BMI, depression, and smoking.

Response:

Thank you for this important observation. We acknowledge this limitation and have highlighted in the limitation section the possibility of over or underestimation of depression based on exclusive use of depression medication as a proxy (Page 18, Lines 66 to 85).

2. Second, the findings therefore represent the mediating role of depression treatment rather than depression itself. To maintain conceptual accuracy, the wording throughout the manuscript (including the Abstract, Introduction, Discussion, and Conclusion) should be revised accordingly.

Response:

We thank you for this comment and have made sweeping changes to show that our study represents the mediating role of depression treatment rather than depression itself in the title and wordings throughout the manuscript.

3. For instance, replacing “participants with depression” with “participants reporting antidepressant use.”

Response:

We have edited all aspects of the paper to show that the use of depression medication was our proxy for depression.

4. Finally, the Discussion and Limitations sections would benefit from a deeper reflection on how this measurement constraint might have influenced the results, such as whether it could have led to an under- or overestimation of the mediation effect.

Response:

Thank you for your comments. We have enriched our discussion and limitation sections highlighting why our use of a proxy for depression may have resulted into over or under-estimation of the depression (Page18, Lines 66-82).

5-Because the study design is cross-sectional, causal inference cannot be made. Although the Methods mention this constraint, the text frequently employs causal expressions (“mediation effect,” “direct effect,” “indirect effect”).
Please revise the manuscript to use neutral terms such as association, link, mediating role, or indirect association.

Response:

Thank for this observation. We have revised the paper using neutral terms as suggested by the reviewer to show that our results have no causal inference.

3-Table 1 incorrectly references “2023 National Youth Tobacco Survey.” It should read “2023 National Health Interview Survey (NHIS)” to remain consistent with the data source cited throughout the manuscript.

Response:

Thank you for noting this observation. We corrected Table 1 to read “2023 National Health Interview Survey (NHIS)” and ensured consistent terminology throughout the manuscript (Page 12, Lines 248-249).

4-The use of a GSEM is a reasonable and well-justified analytical choice for this study. However, the reporting of the model needs to be more detailed to allow readers to fully assess the robustness of the findings. In particular, information on model fit indices such as CFI, TLI, and RMSEA would help clarify how well the model represents the observed data. It would also be useful to specify how the survey’s complex design was accounted for—especially whether sampling weights or clustering adjustments (for example, through the use of survey commands in the analysis software) were applied. Including these details would make the analysis more transparent and easier for other researchers to evaluate or replicate.

Response:

Thank you for this comment. We have edited the reporting of the GSEM to clarify model evaluation. Unlike traditional SEM, generalized structural equation models do not provide global fit indices such as CFI, TLI, or RMSEA, because these chi-square–based measures are not directly applicable to models with non-Gaussian outcomes, nonlinear link functions, or complex survey features. Instead, model assessment in GSEM typically relies on likelihood-based criteria (e.g., AIC and BIC), tests of parameter significance (e.g., Wald tests), and the substantive theoretical justification of the specified pathways. We also incorporated the survey’s complex design, including sampling weights, clustering, and stratification, to ensure appropriate population-level inference. These clarifications have been added to the Methods section (Page 8, Lines 177-181).

This study addresses an important question with an appropriate dataset and analytical method. However, revisions are needed to improve methodological transparency, clarify interpretive boundaries, and comply with ethical reporting standards.

Reviewer #3: While the paper is technically sound and I do not see any major issues in it, I feel that it does not add anything new to the knowledge base. I do not find any particular novelty factor in this and I am afraid that it does not hold the value to be published. Maybe a more regional journal is a better option for this type of paper.

Response:

Thank you for taking the time to review our paper.
