# Supplementary material for: Mediating role of depression medication on association between Body Mass Index and cigarette smoking among US adults: Insights from the NHIS
Source: PLoS One. 2026 Jul 8;21(7):e0351210. doi: 10.1371/journal.pone.0351210 (PMC13345264; doi:10.1371/journal.pone.0351210)
Supplement: Attachment — Submitted filename: Abstract.docx [file pone.0351210.s004.docx]

1. **Abstract – Results Clarity**
   The results reported in the abstract are unclear and lack sufficient quantitative detail. The authors should clearly state the main statistical findings (e.g., direction and magnitude of associations) to improve transparency and consistency with the Results section.
2. **Abstract – Structure and Focus**
   The abstract requires substantial revision to improve coherence. The objectives, methods, results, and conclusions should be more clearly delineated, and redundancy should be minimized.
3. **Keywords**
   Generalized Structural Equation Model should not be included as a keyword. Keywords should reflect the main scientific concepts rather than analytical techniques.
4. **Abbreviations and Terminology**
   All abbreviations (e.g., NCDs) must be defined at first mention and used consistently throughout the manuscript. Inconsistent terminology reduces readability and may confuse readers.
5. **Introduction – Rationale and Novelty**
   The introduction would benefit from a clearer statement of the study’s novelty. The authors should better articulate how this study adds to existing literature and what specific knowledge gap it addresses.
6. **Methods – Variable Definition**
   The definition and operationalization of key variables, particularly depression medication use, should be clarified. The authors should justify the use of medication as a proxy and discuss potential misclassification.
7. **Methods – Statistical Analysis**
   The statistical analysis section requires clearer explanation, particularly regarding model specification, reference groups, and interpretation of direct and indirect associations. This will enhance reproducibility.
8. **Results – Presentation and Consistency**
   The results section needs further refinement. Some findings are difficult to follow, and clearer linkage between text, tables, and figures is required. Redundant reporting should be reduced.
9. **Tables and Figures**
   Tables and figures should be carefully checked for clarity, labeling, and consistency with the text. All abbreviations used in tables should be defined in footnotes.
10. **Discussion – Interpretation of Findings**
    The discussion should more critically interpret the findings rather than restating results. Alternative explanations and comparison with prior studies should be expanded.
11. **Conclusions and Limitations**
    The Conclusions and Limitations sections appear partially duplicated. These sections should be clearly separated, with conclusions summarizing implications and limitations addressing methodological constraints.
12. **Language and Grammar**
    The manuscript contains several grammatical errors and typographical issues. A thorough language edit is recommended to improve clarity and professionalism.
13. **Ethical and Reporting Standards**
    Ethical statements and data descriptions should be reviewed to ensure full compliance with journal guidelines and reporting standards.
