# Supplementary material for: Mediating role of depression medication on association between Body Mass Index and cigarette smoking among US adults: Insights from the NHIS
Source: PLoS One. 2026 Jul 8;21(7):e0351210. doi: 10.1371/journal.pone.0351210 (PMC13345264; doi:10.1371/journal.pone.0351210)
Supplement: Attachment — Submitted filename: Response to Reviewers.docx [file pone.0351210.s006.docx]

**Additional Editor Comments:**

1. Add a sensitivity analysis using the counterfactual framework-based weighting approach (10.1093/aje/kwr525).

Response: Thank you for this valuable suggestion. In response, we conducted an additional sensitivity analysis using a counterfactual mediation framework based on the approach described by Valeri and VanderWeele (2013). Specifically, we implemented a weighting-based causal mediation analysis to estimate natural direct and indirect effects while allowing for treatment-mediator interaction. The results of this analysis are presented in Table S1. Importantly, the findings were consistent with our primary analysis, supporting the robustness of our conclusions.

***Reference:***

Valeri L, Vanderweele TJ. Mediation analysis allowing for exposure-mediator interactions and causal interpretation: theoretical assumptions and implementation with SAS and SPSS macros. Psychol Methods. 2013 Jun;18(2):137-50. doi: 10.1037/a0031034. Epub 2013 Feb 4. Erratum in: Psychol Methods. 2013 Dec;18(4):474. PMID: 23379553; PMCID: PMC3659198.

2. Report the total effect, natural direct effect (NDE), natural indirect effect (NIE), as well as the proportion mediated.

Response: We appreciate this important recommendation. We have expanded our analysis to report the total effect (TE), natural direct effect (NDE), natural indirect effect (NIE), and the proportion mediated (PNIE) within a counterfactual mediation framework. These estimates are presented in Table S1 and summarized in the Results section.

3. Update Figure 1 to depict one exposure, not all the categories of the BMI variable.

Response: Thank you for this helpful suggestion. We have revised Figure 1 to depict a single exposure variable (BMI) rather than multiple category-specific paths. The updated figure now presents a simplified conceptual mediation framework to improve clarity and aligning with standard mediation diagram conventions.

4. The list of potential confounders should be based on expert opinion and literature search. Also, the same set of confounders in both the outcome and mediator models is a very strong assumption that needs more explanation.

Response: Thank you for this important comment. The selection of covariates was informed by prior literature and subject-matter knowledge on the relationships between BMI, depression, and smoking. Sex, race/ethnicity, education, and marital status have been consistently identified as confounders in population-based studies examining these associations, as they are associated with the exposure (BMI), the mediator (depression medication use), and the outcome (current smoking) [Strine et al., 2008; Widome et al., 2009; Wu et al., 2023].

We acknowledge that including the same set of covariates in both the mediator and outcome models is a strong assumption. However, this approach is consistent with standard practice in causal mediation analysis, where a common set of covariates is included in both models to simultaneously address exposure–outcome, mediator–outcome, and exposure–mediator confounding (VanderWeele & Vansteelandt, 2009; Valeri & VanderWeele, 2013; Azagba et al., 2024). We have clarified this rationale in the Methods section of the revised manuscript.

***References:***

VanderWeele, T.J., & Vansteelandt, S. Conceptual issues concerning mediation, interventions and composition. Statistics and Its Interface. 2009; 2, 457-468.

Valeri L, Vanderweele TJ. Mediation analysis allowing for exposure-mediator interactions and causal interpretation: theoretical assumptions and implementation with SAS and SPSS macros. Psychol Methods. 2013 Jun;18(2):137-50. doi: 10.1037/a0031034. Epub 2013 Feb 4. Erratum in: Psychol Methods. 2013 Dec;18(4):474. PMID: 23379553; PMCID: PMC3659198.

Azagba, S., Ebling, T., & Korkmaz, A. Social media and e-cigarette use: The mediating role of mental health conditions. Journal of affective disorders. 2024; 344, 528–534. https://doi.org/10.1016/j.jad.2023.10.053

5. It is unclear whether survey features were utilized in all models.

Response: Thank you for this comment. Yes, survey design features were incorporated in all analyses. Specifically, sampling weights were applied in all models to account for the complex survey design and to ensure nationally representative estimates. We have clarified this in the Methods section of the revised manuscript.

6. Stratify Table 1 by the exposure and report the standardized mean difference (SMD).

Response: We thank you for this crucial suggestion. Table 1 has been revised to present sample characteristics stratified by BMI category (underweight, normal weight, overweight, and obese). Standardized mean differences (SMD) were calculated using Cohen's w (√χ²/N) to quantify the magnitude of differences across BMI groups. The statistical analysis section has been updated to clarify the implementation of survey design features, and Table 1 has been revised accordingly in the manuscript.

7. Please explain why different reference categories were used in Table 2.

Response: Thank you for this comment. The use of different reference categories was intentional to facilitate interpretation of the association across the full BMI spectrum. Model 1 used underweight as the reference category to assess how higher BMI categories differ relative to the lowest BMI group. Model 2 used obese as the reference category to provide complementary comparisons from the opposite end of the BMI distribution. This approach allows for more complete pairwise comparisons across BMI categories without changing the underlying model structure and is consistent with recommendations that the choice of BMI reference category should be explicitly justified and that different reference categories can yield complementary insights (Flegal et al., 2014). We have clarified this in the Table 2 notes and Methods section while citing the rationale.

***References:***

Flegal, K. M., Kit, B. K., & Graubard, B. I. Body mass index categories in observational studies of weight and risk of death. American journal of epidemiology. 2014; 180(3), 288–296. <https://doi.org/10.1093/aje/kwu111>

Pavela, G., Yi, N., Mestre, L., Lartey, S., Xun, P., & Allison, D. B. The associations between relative and absolute body mass index with mortality rate based on predictions from stigma theory. SSM - Population health. 2022; 19, 101200. <https://doi.org/10.1016/j.ssmph.2022.101200>

8. This study’s cross-sectional nature prevents evaluating the temporal relationship between the exposure, mediator and outcome. That should be thoroughly discussed.

Response: We appreciate this important point. We agree that the cross-sectional nature of the data is a significant limitation that requires thorough discussion. We have expanded the limitations section to explicitly address the inability to establish temporal ordering between BMI, depression medication use, and smoking status, and have discussed the implications of this limitation for the interpretation of the mediation pathways.

Reviewer #1:

1. Abstract – Results Clarity
The results reported in the abstract are unclear and lack sufficient quantitative detail. The authors should clearly state the main statistical findings (e.g., direction and magnitude of associations) to improve transparency and consistency with the Results section.

Response: Thank you for your comments. We have revised the abstract section by adding some details about the direction and magnitude of association of the main statistical findings within the text limit for the abstract section.

2. Abstract – Structure and Focus
The abstract requires substantial revision to improve coherence. The objectives, methods, results, and conclusions should be more clearly delineated, and redundancy should be minimized.

Response: Thank you for your comments. We have revised the text of the abstract section to highlight the objectives, methods and results and conclusion in a clearer manner.
3. Keywords
Generalized Structural Equation Model should not be included as a keyword. Keywords should reflect the main scientific concepts rather than analytical techniques.

Responses: Thank you for your comments. We have deleted Generalized Structural Equation Model from our keyword list as advised.

4. Abbreviations and Terminology
All abbreviations (e.g., NCDs) must be defined at first mention and used consistently throughout the manuscript. Inconsistent terminology reduces readability and may confuse readers.

Response: Thank you for your comments. We have edited the text to so that acronyms are defined on first mention. We have also worked on consistency with the use of terms throughout the text to improve its readability.

5. Introduction – Rationale and Novelty
The introduction would benefit from a clearer statement of the study’s novelty. The authors should better articulate how this study adds to existing literature and what specific knowledge gap it addresses.

Response: Thank you for your comments. Our study is unique because it assesses the effect of a moderator (i.e, use of depression medication) on cigarette smoking across various Basal Metabolic Index (BMI) categories classified in our study (underweight, healthy weight, overweight and obese. Previous studies have mainly focused on whether participants were obese or not whereas our study provides insight into the relationship between depression medication use and cigarette smoking across all the BMI categories. An understanding of this role is important in planning cigarette smoking cessation interventions in health care settings where clients may be experiencing depression.

6. Methods – Variable Definition
The definition and operationalization of key variables, particularly depression medication use, should be clarified. The authors should justify the use of medication as a proxy and discuss potential misclassification.

Responses: We used depression medication as a proxy for diagnosis of depression among respondents because of the unavailability of data on depression on the NHIS dataset. The potential for misclassification has been highlighted in the limitations section in which we note that some individuals may have depression without being prescribed depression medication while also excluding people receiving non-pharmacological interventions to treat their depression.

7. Methods – Statistical Analysis
The statistical analysis section requires clearer explanation, particularly regarding model specification, reference groups, and interpretation of direct and indirect associations. This will enhance reproducibility.

Response: Thank you for your comments. We have revised the statistical analysis text of the Materials and Methods section to highlight the specification of our models, the reference groups used, and interpretation of direct and indirect associations.

8. Results – Presentation and Consistency
The results section needs further refinement. Some findings are difficult to follow, and clearer linkage between text, tables, and figures is required. Redundant reporting should be reduced.

Response: Thank you for your comments. We have edited the text of the results section linking the text to the tables. We have also eliminated redundant phrases in reporting our results.

9. Tables and Figures
Tables and figures should be carefully checked for clarity, labeling, and consistency with the text. All abbreviations used in tables should be defined in footnotes.

Response: Thank you for your comments. We have redrawn Tables 1 and 2 to ensure greater clarity of the information and reduced labelling errors. We have also labelled each table with all acronyms defined either in the body of the table or in the footnotes.

10. Discussion – Interpretation of Findings
The discussion should more critically interpret the findings rather than restating results. Alternative explanations and comparison with prior studies should be expanded.

Response: Thank you for your comments. We have edited the Discussion section to better compare our findings with that obtained in other studies and provided explanations to why some of our results either agree or contrast with that which was obtained elsewhere.

11. Conclusions and Limitations
The Conclusions and Limitations sections appear partially duplicated. These sections should be clearly separated, with conclusions summarizing implications and limitations addressing methodological constraints.

Response: Thank you for your observation. We have relabeled the Conclusions section correctly and edited the Limitations section highlighting the methodological constraints of our study and suggesting how future studies can improve on our methodology.

12. Language and Grammar
The manuscript contains several grammatical errors and typographical issues. A thorough language edit is recommended to improve clarity and professionalism.

Response: Thank you for your comments. We have taken time to edit the text to correct grammatical errors and limit typographical issues.

13. Ethical and Reporting Standards
Ethical statements and data descriptions should be reviewed to ensure full compliance with journal guidelines and reporting standards.

Response: Thank you for your comments. We have reviewed our ethical statement and it appears to be in line with the requirements of the PLOS One journal.
