# Supplementary material for: Mediating role of depression medication on association between Body Mass Index and cigarette smoking among US adults: Insights from the NHIS
Source: PLoS One. 2026 Jul 8;21(7):e0351210. doi: 10.1371/journal.pone.0351210 (PMC13345264; doi:10.1371/journal.pone.0351210)
Supplement: Peer review history

# Peer review: Decision Letter 0

**DOI:** 10.1371/journal.pone.0351210.r001

**Authors:** David Adedia

29 Oct 2025

PONE-D-25-35034

Mediation effect of depression on association between Body Mass Index and cigarette smoking among US adults: insights from the NHIS

PLOS ONE

Dear Dr. Onigbogi,

Thank you for submitting your manuscript to PLOS ONE. After careful consideration, we feel that it has merit but does not fully meet PLOS ONE’s publication criteria as it currently stands. Therefore, we invite you to submit a revised version of the manuscript that addresses the points raised during the review process.

==============================

**ACADEMIC EDITOR:**

The authors used Generalized Structural Equation Model to ascertain the mediation effects depression on the relationship between BMI and cigarette smoking. However, the authors did not provide detailed explanation of this methodology as well as sources. How depression was measured is problematic and the authors should use the appropriate measure in the study. The study lacked conceptual framework, and the authors should provide this for the relationships in the variables. These comments in addition to the reviewers’ comments should be addressed specifically to improve the current state of the manuscript.

==============================

Please submit your revised manuscript by Dec 13 2025 11:59PM. If you will need more time than this to complete your revisions, please reply to this message or contact the journal office at plosone@plos.org. When you're ready to submit your revision, log on to https://www.editorialmanager.com/pone/ and select the 'Submissions Needing Revision' folder to locate your manuscript file.

Please include the following items when submitting your revised manuscript:

- A rebuttal letter that responds to each point raised by the academic editor and reviewer(s). You should upload this letter as a separate file labeled 'Response to Reviewers'.
- A marked-up copy of your manuscript that highlights changes made to the original version. You should upload this as a separate file labeled 'Revised Manuscript with Track Changes'.
- An unmarked version of your revised paper without tracked changes. You should upload this as a separate file labeled 'Manuscript'.

If you would like to make changes to your financial disclosure, please include your updated statement in your cover letter. Guidelines for resubmitting your figure files are available below the reviewer comments at the end of this letter.

If applicable, we recommend that you deposit your laboratory protocols in protocols.io to enhance the reproducibility of your results. Protocols.io assigns your protocol its own identifier (DOI) so that it can be cited independently in the future. For instructions see: https://journals.plos.org/plosone/s/submission-guidelines#loc-laboratory-protocols. Additionally, PLOS ONE offers an option for publishing peer-reviewed Lab Protocol articles, which describe protocols hosted on protocols.io. Read more information on sharing protocols at https://plos.org/protocols?utm_medium=editorial-email&utm_source=authorletters&utm_campaign=protocols.

We look forward to receiving your revised manuscript.

Kind regards,

David Adedia, Ph.D

Academic Editor

PLOS ONE

Journal Requirements:

When submitting your revision, we need you to address these additional requirements.

1. Please ensure that your manuscript meets PLOS ONE's style requirements, including those for file naming. The PLOS ONE style templates can be found at

https://journals.plos.org/plosone/s/file?id=wjVg/PLOSOne_formatting_sample_main_body.pdf and

https://journals.plos.org/plosone/s/file?id=ba62/PLOSOne_formatting_sample_title_authors_affiliations.pdf.

2. Please include a caption for figure 1.

3. If the reviewer comments include a recommendation to cite specific previously published works, please review and evaluate these publications to determine whether they are relevant and should be cited. There is no requirement to cite these works unless the editor has indicated otherwise.

[Note: HTML markup is below. Please do not edit.]

Reviewers' comments:

Reviewer's Responses to Questions

**Comments to the Author**

1. Is the manuscript technically sound, and do the data support the conclusions?

The manuscript must describe a technically sound piece of scientific research with data that supports the conclusions. Experiments must have been conducted rigorously, with appropriate controls, replication, and sample sizes. The conclusions must be drawn appropriately based on the data presented.

Reviewer #1: Yes

Reviewer #2: Partly

Reviewer #3: Yes

**********

2. Has the statistical analysis been performed appropriately and rigorously?

Reviewer #1: Yes

Reviewer #2: I Don't Know

Reviewer #3: Yes

**********

3. Have the authors made all data underlying the findings in their manuscript fully available?

The PLOS Data policy requires authors to make all data underlying the findings described in their manuscript fully available without restriction, with rare exception (please refer to the Data Availability Statement in the manuscript PDF file). The data should be provided as part of the manuscript or its supporting information, or deposited to a public repository. For example, in addition to summary statistics, the data points behind means, medians and variance measures should be available. If there are restrictions on publicly sharing data—e.g. participant privacy or use of data from a third party—those must be specified.

Reviewer #1: No

Reviewer #2: Yes

Reviewer #3: Yes

**********

4. Is the manuscript presented in an intelligible fashion and written in standard English?

PLOS ONE does not copyedit accepted manuscripts, so the language in submitted articles must be clear, correct, and unambiguous. Any typographical or grammatical errors should be corrected at revision, so please note any specific errors here.

Reviewer #1: Yes

Reviewer #2: Yes

Reviewer #3: Yes

**********

5. Review Comments to the Author

Please use the space provided to explain your answers to the questions above. You may also include additional comments for the author, including concerns about dual publication, research ethics, or publication ethics. (Please upload your review as an attachment if it exceeds 20,000 characters)

Reviewer #1: . Conceptual Clarity of Mediation Framework

• The manuscript explores depression as a mediator between BMI and smoking, but the theoretical rationale for this pathway needs clearer justification.

• Recommendation: Strengthen the conceptual model in the Introduction by explaining why depression might mediate the BMI–smoking link, not just correlate with both. Consider citing behavioral or neurobiological mechanisms that support this mediation.

2. Measurement of Depression

• Depression is measured via a single binary item: use of prescription medication. This may not capture the full spectrum of depressive symptoms or severity.

• Recommendation: Acknowledge this limitation explicitly in the Discussion. Consider discussing how this proxy may underestimate or misclassify depression, and suggest future studies use validated multi-item scales.

3. Interpretation of Mediation Effects

• The manuscript reports odds ratios for indirect effects but does not clearly explain how these were calculated or interpreted.

• Recommendation: Clarify the mediation analysis framework (e.g., product of coefficients, delta method) and explain how indirect effects were derived and interpreted in the context of GSEM.

4. Model Specification and Reference Groups

• Two models are used with different BMI reference categories (underweight and obese), but the rationale for this dual approach is not fully explained.

• Recommendation: Justify the use of dual reference groups in the Methods and clarify how this enhances interpretability or addresses clinical relevance.

5. Generalizability and Sample Description

• The sample is drawn from NHIS, but the manuscript inconsistently refers to it as the “National Youth Tobacco Survey” in Table 1.

• Recommendation: Correct this labeling and ensure consistent terminology throughout. Also, discuss generalizability given the cross-sectional design and self-reported data.

6. Ethical Statement and Data Access

• The ethics section states that IRB approval was not required due to public data use, but this should be framed more formally.

• Recommendation: Rephrase to: “This study used publicly available, de-identified data from the NHIS. Ethical approval was not required per institutional guidelines.”

7. Language and Formatting

• Several sections contain grammatical errors, inconsistent tense, and formatting issues (e.g., spacing, punctuation, equation formatting).

• Recommendation: Conduct a thorough language and style edit before resubmission. Ensure equations are properly typeset and tables are consistently labeled.

8. Formatting

• Check the plos one journal instruction

Reviewer #2: Dear Authors,

This manuscript investigates the mediating role of depression in the association between BMI and cigarette smoking using the 2023 National Health Interview Survey (NHIS). The topic is of high relevance to public health, and the inclusion of all BMI categories (underweight, normal, overweight, obese) provides valuable detail. The use of a GSEM strengthens the methodological framework. However, several key methodological and interpretive limitations must be addressed before the manuscript can be considered technically sound. This review supports a Major Revision recommendation.

1-The primary methodological concern pertains to the measurement of depression. In this study, depression was identified solely through participants’ self-reported use of prescribed antidepressant medication. This approach does not capture the full spectrum of depressive symptoms; rather, it reflects only individuals currently receiving pharmacological treatment.

As a result, several issues arise. First, it likely excludes people with undiagnosed or untreated depression, those receiving non-pharmacological interventions such as psychotherapy, or individuals with limited access to mental health care. This selective definition could bias the observed associations between BMI, depression, and smoking.

Second, the findings therefore represent the mediating role of depression treatment rather than depression itself. To maintain conceptual accuracy, the wording throughout the manuscript (including the Abstract, Introduction, Discussion, and Conclusion) should be revised accordingly. For instance, replacing “participants with depression” with “participants reporting antidepressant use.”

Finally, the Discussion and Limitations sections would benefit from a deeper reflection on how this measurement constraint might have influenced the results, such as whether it could have led to an under- or overestimation of the mediation effect.

2-Because the study design is cross-sectional, causal inference cannot be made. Although the Methods mention this constraint, the text frequently employs causal expressions (“mediation effect,” “direct effect,” “indirect effect”).

Please revise the manuscript to use neutral terms such as association, link, mediating role, or indirect association.

3-Table 1 incorrectly references “2023 National Youth Tobacco Survey.” It should read “2023 National Health Interview Survey (NHIS)” to remain consistent with the data source cited throughout the manuscript.

4-The use of a GSEM is a reasonable and well-justified analytical choice for this study. However, the reporting of the model needs to be more detailed to allow readers to fully assess the robustness of the findings. In particular, information on model fit indices such as CFI, TLI, and RMSEA would help clarify how well the model represents the observed data. It would also be useful to specify how the survey’s complex design was accounted for—especially whether sampling weights or clustering adjustments (for example, through the use of survey commands in the analysis software) were applied. Including these details would make the analysis more transparent and easier for other researchers to evaluate or replicate.

This study addresses an important question with an appropriate dataset and analytical method. However, revisions are needed to improve methodological transparency, clarify interpretive boundaries, and comply with ethical reporting standards.

Reviewer #3: While the paper is technically sound and I do not see any major issues in it, I feel that it does not add anything new to the knowledge base. I do not find any particular novelty factor in this and I am afraid that it does not hold the value to be published. Maybe a more regional journal is a better option for this type of paper.

**********

6. PLOS authors have the option to publish the peer review history of their article (what does this mean?). If published, this will include your full peer review and any attached files.

If you choose “no”, your identity will remain anonymous but your review may still be made public.

**Do you want your identity to be public for this peer review?**  For information about this choice, including consent withdrawal, please see our Privacy Policy.

Reviewer #1: No

Reviewer #2: No

Reviewer #3: No

**********

[NOTE: If reviewer comments were submitted as an attachment file, they will be attached to this email and accessible via the submission site. Please log into your account, locate the manuscript record, and check for the action link "View Attachments". If this link does not appear, there are no attachment files.]

To ensure your figures meet our technical requirements, please review our figure guidelines: https://journals.plos.org/plosone/s/figures

You may also use PLOS’s free figure tool, NAAS, to help you prepare publication quality figures: https://journals.plos.org/plosone/s/figures#loc-tools-for-figure-preparation.

NAAS will assess whether your figures meet our technical requirements by comparing each figure against our figure specifications.

Attachment Submitted filename: comment.docx

# Author comment: Author response to Decision Letter 1

**DOI:** 10.1371/journal.pone.0351210.r002

17 Dec 2025

Response to Editor and reviewers

Dear Editor,

Thank you for the opportunity to review and submit our paper once again to PLOS One journal taking into consideration the response from the reviewers.

In addition, we have changed the title to ‘Mediating role of depression medication on association between Body Mass Index and cigarette smoking among US adults: Insights from the NHIS’ to reflect the comments by reviewers.

The authors have made the changes as suggested by the reviewers. Our responses are highlighted below.

Thank you.

Olanrewaju Onigbogi

09 December 2025

Reviewer #1: Conceptual Clarity of Mediation Framework

1. The manuscript explores depression as a mediator between BMI and smoking, but the theoretical rationale for this pathway needs clearer justification.

• Recommendation: Strengthen the conceptual model in the Introduction by explaining why depression might mediate the BMI–smoking link, not just correlate with both. Consider citing behavioral or neurobiological mechanisms that support this mediation.

Response:

Thank you for this observation. To further strengthen the conceptual model for our study, we added some detail about how depression mediates the link between BMI and smoking through shared neurobiological reward pathways, maladaptive coping behaviors, and chronic inflammation (Page 4, Lines 92-95).

2. Measurement of Depression

• Depression is measured via a single binary item: use of prescription medication. This may not capture the full spectrum of depressive symptoms or severity.

• Recommendation: Acknowledge this limitation explicitly in the Discussion. Consider discussing how this proxy may underestimate or misclassify depression, and suggest future studies use validated multi-item scales.

Response:

Thank you for the comments. We have acknowledged this limitation in the discussion highlighting why using depression medication as a proxy may underestimate or misclassify depression. We have also suggested that future studies use validated scales such as the Patient Health Questionnaire-9 (PHQ-9) for self-report screening, the clinician-rated Hamilton Depression Rating Scale (HAM-D), and the Beck Depression Inventory (BDI) – (Page 18, Lines 66-82).

3. Interpretation of Mediation Effects

• The manuscript reports odds ratios for indirect effects but does not clearly explain how these were calculated or interpreted.

• Recommendation: Clarify the mediation analysis framework (e.g., product of coefficients, delta method) and explain how indirect effects were derived and interpreted in the context of GSEM.

Response:

Thank you for this important comment. We agree that the original description of the mediation framework needed additional clarification. In the revised manuscript, we expanded the Methods section under ‘Statistical Analysis’ to explain how indirect association were computed in the GSEM framework and how odds ratios were derived and interpreted (Page 8, Lines 160-171).

4. Model Specification and Reference Groups

• Two models are used with different BMI reference categories (underweight and obese), but the rationale for this dual approach is not fully explained.

• Recommendation: Justify the use of dual reference groups in the Methods and clarify how this enhances interpretability or addresses clinical relevance.

Response:

Thank you for this comment. We clarified the rationale for using two BMI reference groups. Methodological guidance supports using alternative reference categories to improve interpretability of categorical predictors (Hosmer & Lemeshow, 2013; Kleinbaum & Klein, 2010; Long & Freese, 2014). Because underweight and obese represent clinically distinct extremes of the BMI spectrum, analyzing both as reference groups provides meaningful contrasts in both directions and avoids a one-sided comparison (Nuttall, 2015; Flegal et al., 2013). This explanation has been added to the Methods section (Page 9, Lines 185-196).

References:

Hosmer DW, Lemeshow S, Sturdivant RX. Applied Logistic Regression. 3rd ed. Wiley; 2013.

Kleinbaum DG, Klein M. Logistic Regression: A Self-Learning Text. 3rd ed. Springer; 2010.

Long JS, Freese J. Regression Models for Categorical Dependent Variables Using Stata. 3rd ed. College Station, TX: Stata Press; 2014.

Nuttall FQ. Body Mass Index: Obesity, BMI, and Health: A Critical Review. Nutr Today. 2015;50(3):117–128.

Flegal KM, Kit BK, Orpana H, Graubard BI. Association of All-Cause Mortality With Overweight and Obesity Using Standard Body Mass Index Categories: A Systematic Review and Meta-analysis. JAMA. 2013;309(1):71–82.

5. Generalizability and Sample Description

• The sample is drawn from NHIS, but the manuscript inconsistently refers to it as the “National Youth Tobacco Survey” in Table 1.

• Recommendation: Correct this labeling and ensure consistent terminology throughout. Also, discuss generalizability given the cross-sectional design and self-reported data.

Response:

Thank you for noting this inconsistency. We corrected the labeling in Table 1, replacing “National Youth Tobacco Survey” with the correct data source, the National Health Interview Survey (NHIS), and reviewed the manuscript to ensure consistent terminology throughout (Page12).

We also added a brief statement on generalizability, noting that findings are limited by the cross-sectional design and reliance on self-reported measures, which may introduce reporting bias and prevent causal inference. This clarification has been added to the Discussion section (Page 16, Lines 25-40).

6. Ethical Statement and Data Access

• The ethics section states that IRB approval was not required due to public data use, but this should be framed more formally.

• Recommendation: Rephrase to: “This study used publicly available, de-identified data from the NHIS. Ethical approval was not required per institutional guidelines.”

Response:

Thank you for this observation and do agree that the suggested text is a better expression of what we did. We have quoted the suggested phrase verbatim and added to the text in the Ethical compliance section (Page 6, Lines 131-132)

7. Language and Formatting

• Several sections contain grammatical errors, inconsistent tense, and formatting issues (e.g., spacing, punctuation, equation formatting).

• Recommendation: Conduct a thorough language and style edit before resubmission. Ensure equations are properly typeset and tables are consistently labeled.

Response:

Thank you for your observation. We have edited the document to address grammatical errors, inconsistent tense, and formatting issues. We have also redone the equation and tables (Page 8, Lines 121-126, Page 12 Lines 248-249).

8. Formatting

• Check the plos one journal instruction

Response:

Thank you for your comments. We have revisited the POLS One site addressing formatting issues in line with instructions.

Reviewer #2: Dear Authors,

This manuscript investigates the mediating role of depression in the association between BMI and cigarette smoking using the 2023 National Health Interview Survey (NHIS). The topic is of high relevance to public health, and the inclusion of all BMI categories (underweight, normal, overweight, obese) provides valuable detail. The use of a GSEM strengthens the methodological framework. However, several key methodological and interpretive limitations must be addressed before the manuscript can be considered technically sound. This review supports a Major Revision recommendation.

1-The primary methodological concern pertains to the measurement of depression. In this study, depression was identified solely through participants’ self-reported use of prescribed antidepressant medication. This approach does not capture the full spectrum of depressive symptoms; rather, it reflects only individuals currently receiving pharmacological treatment.

As a result, several issues arise. First, it likely excludes people with undiagnosed or untreated depression, those receiving non-pharmacological interventions such as psychotherapy, or individuals with limited access to mental health care. This selective definition could bias the observed associations between BMI, depression, and smoking.

Response:

Thank you for this important observation. We acknowledge this limitation and have highlighted in the limitation section the possibility of over or underestimation of depression based on exclusive use of depression medication as a proxy (Page 18, Lines 66 to 85).

2. Second, the findings therefore represent the mediating role of depression treatment rather than depression itself. To maintain conceptual accuracy, the wording throughout the manuscript (including the Abstract, Introduction, Discussion, and Conclusion) should be revised accordingly.

Response:

We thank you for this comment and have made sweeping changes to show that our study represents the mediating role of depression treatment rather than depression itself in the title and wordings throughout the manuscript.

3. For instance, replacing “participants with depression” with “participants reporting antidepressant use.”

Response:

We have edited all aspects of the paper to show that the use of depression medication was our proxy for depression.

4. Finally, the Discussion and Limitations sections would benefit from a deeper reflection on how this measurement constraint might have influenced the results, such as whether it could have led to an under- or overestimation of the mediation effect.

Response:

Thank you for your comments. We have enriched our discussion and limitation sections highlighting why our use of a proxy for depression may have resulted into over or under-estimation of the depression (Page18, Lines 66-82).

5-Because the study design is cross-sectional, causal inference cannot be made. Although the Methods mention this constraint, the text frequently employs causal expressions (“mediation effect,” “direct effect,” “indirect effect”).

Please revise the manuscript to use neutral terms such as association, link, mediating role, or indirect association.

Response:

Thank for this observation. We have revised the paper using neutral terms as suggested by the reviewer to show that our results have no causal inference.

3-Table 1 incorrectly references “2023 National Youth Tobacco Survey.” It should read “2023 National Health Interview Survey (NHIS)” to remain consistent with the data source cited throughout the manuscript.

Response:

Thank you for noting this observation. We corrected Table 1 to read “2023 National Health Interview Survey (NHIS)” and ensured consistent terminology throughout the manuscript (Page 12, Lines 248-249).

4-The use of a GSEM is a reasonable and well-justified analytical choice for this study. However, the reporting of the model needs to be more detailed to allow readers to fully assess the robustness of the findings. In particular, information on model fit indices such as CFI, TLI, and RMSEA would help clarify how well the model represents the observed data. It would also be useful to specify how the survey’s complex design was accounted for—especially whether sampling weights or clustering adjustments (for example, through the use of survey commands in the analysis software) were applied. Including these details would make the analysis more transparent and easier for other researchers to evaluate or replicate.

Response:

Thank you for this comment. We have edited the reporting of the GSEM to clarify model evaluation. Unlike traditional SEM, generalized structural equation models do not provide global fit indices such as CFI, TLI, or RMSEA, because these chi-square–based measures are not directly applicable to models with non-Gaussian outcomes, nonlinear link functions, or complex survey features. Instead, model assessment in GSEM typically relies on likelihood-based criteria (e.g., AIC and BIC), tests of parameter significance (e.g., Wald tests), and the substantive theoretical justification of the specified pathways. We also incorporated the survey’s complex design, including sampling weights, clustering, and stratification, to ensure appropriate population-level inference. These clarifications have been added to the Methods section (Page 8, Lines 177-181).

This study addresses an important question with an appropriate dataset and analytical method. However, revisions are needed to improve methodological transparency, clarify interpretive boundaries, and comply with ethical reporting standards.

Reviewer #3: While the paper is technically sound and I do not see any major issues in it, I feel that it does not add anything new to the knowledge base. I do not find any particular novelty factor in this and I am afraid that it does not hold the value to be published. Maybe a more regional journal is a better option for this type of paper.

Response:

Thank you for taking the time to review our paper.

Attachment Submitted filename: 11th Dec Response to Reviewers.docx

# Peer review: Decision Letter 1

**DOI:** 10.1371/journal.pone.0351210.r003

**Authors:** Belal Hossain

8 Mar 2026

PONE-D-25-35034R1

Mediating role of depression medication on association between Body Mass Index and cigarette smoking among US adults: Insights from the NHIS

PLOS One

Dear Dr. Onigbogi,

Thank you for submitting your manuscript to PLOS ONE. After careful consideration, we feel that it has merit but does not fully meet PLOS ONE’s publication criteria as it currently stands. Therefore, we invite you to submit a revised version of the manuscript that addresses the points raised during the review process.

Please submit your revised manuscript by Apr 22 2026 11:59PM. If you will need more time than this to complete your revisions, please reply to this message or contact the journal office at plosone@plos.org. When you're ready to submit your revision, log on to https://www.editorialmanager.com/pone/ and select the 'Submissions Needing Revision' folder to locate your manuscript file.

Please include the following items when submitting your revised manuscript:

- A letter that responds to each point raised by the academic editor and reviewer(s). You should upload this letter as a separate file labeled 'Response to Reviewers'.
- A marked-up copy of your manuscript that highlights changes made to the original version. You should upload this as a separate file labeled 'Revised Manuscript with Track Changes'.
- An unmarked version of your revised paper without tracked changes. You should upload this as a separate file labeled 'Manuscript'.

If you would like to make changes to your financial disclosure, please include your updated statement in your cover letter. Guidelines for resubmitting your figure files are available below the reviewer comments at the end of this letter.

If applicable, we recommend that you deposit your laboratory protocols in protocols.io to enhance the reproducibility of your results. Protocols.io assigns your protocol its own identifier (DOI) so that it can be cited independently in the future. For instructions see: https://journals.plos.org/plosone/s/submission-guidelines#loc-laboratory-protocols. Additionally, PLOS ONE offers an option for publishing peer-reviewed Lab Protocol articles, which describe protocols hosted on protocols.io. Read more information on sharing protocols at https://plos.org/protocols?utm_medium=editorial-email&utm_source=authorletters&utm_campaign=protocols.

We look forward to receiving your revised manuscript.

Kind regards,

Belal Hossain, PhD

Academic Editor

PLOS One

Journal Requirements:

If the reviewer comments include a recommendation to cite specific previously published works, please review and evaluate these publications to determine whether they are relevant and should be cited. There is no requirement to cite these works unless the editor has indicated otherwise.

Additional Editor Comments:

1. Add a sensitivity analysis using the counterfactual framework-based weighting approach (10.1093/aje/kwr525).

2. Report the total effect,  natural direct effect (NDE), natural indirect effect (NIE), as well as the proportion mediated.

3. Update Figure 1 to depict one exposure, not all the categories of the BMI variable.

4. The list of potential confounders should be based on expert opinion and literature search. Also, the same set of confounders in both the outcome and mediator models is a very strong assumption that needs more explanation.

5. It is unclear whether survey features were utilized in all models.

6. Stratify Table 1 by the exposure and report the standardized mean difference (SMD).

7. Please explain why different reference categories were used in Table 2.

8. This study’s cross-sectional nature prevents evaluating the temporal relationship between the exposure, mediator and outcome. That should be thoroughly discussed.

[Note: HTML markup is below. Please do not edit.]

Reviewers' comments:

Reviewer's Responses to Questions

**Comments to the Author**

1. If the authors have adequately addressed your comments raised in a previous round of review and you feel that this manuscript is now acceptable for publication, you may indicate that here to bypass the “Comments to the Author” section, enter your conflict of interest statement in the “Confidential to Editor” section, and submit your "Accept" recommendation.

Reviewer #1: (No Response)

Reviewer #3: All comments have been addressed

**********

2. Is the manuscript technically sound, and do the data support the conclusions?

The manuscript must describe a technically sound piece of scientific research with data that supports the conclusions. Experiments must have been conducted rigorously, with appropriate controls, replication, and sample sizes. The conclusions must be drawn appropriately based on the data presented.

Reviewer #1: (No Response)

Reviewer #3: Yes

**********

3. Has the statistical analysis been performed appropriately and rigorously?

Reviewer #1: (No Response)

Reviewer #3: Yes

**********

4. Have the authors made all data underlying the findings in their manuscript fully available?

The PLOS Data policy requires authors to make all data underlying the findings described in their manuscript fully available without restriction, with rare exception (please refer to the Data Availability Statement in the manuscript PDF file). The data should be provided as part of the manuscript or its supporting information, or deposited to a public repository. For example, in addition to summary statistics, the data points behind means, medians and variance measures should be available. If there are restrictions on publicly sharing data—e.g. participant privacy or use of data from a third party—those must be specified.

Reviewer #1: (No Response)

Reviewer #3: Yes

**********

5. Is the manuscript presented in an intelligible fashion and written in standard English?

PLOS ONE does not copyedit accepted manuscripts, so the language in submitted articles must be clear, correct, and unambiguous. Any typographical or grammatical errors should be corrected at revision, so please note any specific errors here.

Reviewer #1: (No Response)

Reviewer #3: Yes

**********

6. Review Comments to the Author

Please use the space provided to explain your answers to the questions above. You may also include additional comments for the author, including concerns about dual publication, research ethics, or publication ethics. (Please upload your review as an attachment if it exceeds 20,000 characters)

Reviewer #1: 1. Abstract – Results Clarity

The results reported in the abstract are unclear and lack sufficient quantitative detail. The authors should clearly state the main statistical findings (e.g., direction and magnitude of associations) to improve transparency and consistency with the Results section.

2. Abstract – Structure and Focus

The abstract requires substantial revision to improve coherence. The objectives, methods, results, and conclusions should be more clearly delineated, and redundancy should be minimized.

3. Keywords

Generalized Structural Equation Model should not be included as a keyword. Keywords should reflect the main scientific concepts rather than analytical techniques.

4. Abbreviations and Terminology

All abbreviations (e.g., NCDs) must be defined at first mention and used consistently throughout the manuscript. Inconsistent terminology reduces readability and may confuse readers.

5. Introduction – Rationale and Novelty

The introduction would benefit from a clearer statement of the study’s novelty. The authors should better articulate how this study adds to existing literature and what specific knowledge gap it addresses.

6. Methods – Variable Definition

The definition and operationalization of key variables, particularly depression medication use, should be clarified. The authors should justify the use of medication as a proxy and discuss potential misclassification.

7. Methods – Statistical Analysis

The statistical analysis section requires clearer explanation, particularly regarding model specification, reference groups, and interpretation of direct and indirect associations. This will enhance reproducibility.

8. Results – Presentation and Consistency

The results section needs further refinement. Some findings are difficult to follow, and clearer linkage between text, tables, and figures is required. Redundant reporting should be reduced.

9. Tables and Figures

Tables and figures should be carefully checked for clarity, labeling, and consistency with the text. All abbreviations used in tables should be defined in footnotes.

10. Discussion – Interpretation of Findings

The discussion should more critically interpret the findings rather than restating results. Alternative explanations and comparison with prior studies should be expanded.

11. Conclusions and Limitations

The Conclusions and Limitations sections appear partially duplicated. These sections should be clearly separated, with conclusions summarizing implications and limitations addressing methodological constraints.

12. Language and Grammar

The manuscript contains several grammatical errors and typographical issues. A thorough language edit is recommended to improve clarity and professionalism.

13. Ethical and Reporting Standards

Ethical statements and data descriptions should be reviewed to ensure full compliance with journal guidelines and reporting standards.

Reviewer #3: Good work. I can see that you have carefully incorporated all the suggestions, which have been given by the reviewers.

**********

7. PLOS authors have the option to publish the peer review history of their article (what does this mean?). If published, this will include your full peer review and any attached files.

If you choose “no”, your identity will remain anonymous but your review may still be made public.

**Do you want your identity to be public for this peer review?**  For information about this choice, including consent withdrawal, please see our Privacy Policy.

Reviewer #1: No

Reviewer #3: No

**********

[NOTE: If reviewer comments were submitted as an attachment file, they will be attached to this email and accessible via the submission site. Please log into your account, locate the manuscript record, and check for the action link "View Attachments". If this link does not appear, there are no attachment files.]

To ensure your figures meet our technical requirements, please review our figure guidelines: https://journals.plos.org/plosone/s/figures

You may also use PLOS’s free figure tool, NAAS, to help you prepare publication quality figures: https://journals.plos.org/plosone/s/figures#loc-tools-for-figure-preparation.

NAAS will assess whether your figures meet our technical requirements by comparing each figure against our figure specifications.

Attachment Submitted filename: Abstract.docx

# Author comment: Author response to Decision Letter 2

**DOI:** 10.1371/journal.pone.0351210.r004

6 May 2026

Additional Editor Comments:

1. Add a sensitivity analysis using the counterfactual framework-based weighting approach (10.1093/aje/kwr525).

Response: Thank you for this valuable suggestion. In response, we conducted an additional sensitivity analysis using a counterfactual mediation framework based on the approach described by Valeri and VanderWeele (2013). Specifically, we implemented a weighting-based causal mediation analysis to estimate natural direct and indirect effects while allowing for treatment-mediator interaction. The results of this analysis are presented in Table S1. Importantly, the findings were consistent with our primary analysis, supporting the robustness of our conclusions.

Reference:

Valeri L, Vanderweele TJ. Mediation analysis allowing for exposure-mediator interactions and causal interpretation: theoretical assumptions and implementation with SAS and SPSS macros. Psychol Methods. 2013 Jun;18(2):137-50. doi: 10.1037/a0031034. Epub 2013 Feb 4. Erratum in: Psychol Methods. 2013 Dec;18(4):474. PMID: 23379553; PMCID: PMC3659198.

2. Report the total effect, natural direct effect (NDE), natural indirect effect (NIE), as well as the proportion mediated.

Response: We appreciate this important recommendation. We have expanded our analysis to report the total effect (TE), natural direct effect (NDE), natural indirect effect (NIE), and the proportion mediated (PNIE) within a counterfactual mediation framework. These estimates are presented in Table S1 and summarized in the Results section.

3. Update Figure 1 to depict one exposure, not all the categories of the BMI variable.

Response: Thank you for this helpful suggestion. We have revised Figure 1 to depict a single exposure variable (BMI) rather than multiple category-specific paths. The updated figure now presents a simplified conceptual mediation framework to improve clarity and aligning with standard mediation diagram conventions.

4. The list of potential confounders should be based on expert opinion and literature search. Also, the same set of confounders in both the outcome and mediator models is a very strong assumption that needs more explanation.

Response: Thank you for this important comment. The selection of covariates was informed by prior literature and subject-matter knowledge on the relationships between BMI, depression, and smoking. Sex, race/ethnicity, education, and marital status have been consistently identified as confounders in population-based studies examining these associations, as they are associated with the exposure (BMI), the mediator (depression medication use), and the outcome (current smoking) [Strine et al., 2008; Widome et al., 2009; Wu et al., 2023].

We acknowledge that including the same set of covariates in both the mediator and outcome models is a strong assumption. However, this approach is consistent with standard practice in causal mediation analysis, where a common set of covariates is included in both models to simultaneously address exposure–outcome, mediator–outcome, and exposure–mediator confounding (VanderWeele & Vansteelandt, 2009; Valeri & VanderWeele, 2013; Azagba et al., 2024). We have clarified this rationale in the Methods section of the revised manuscript.

References:

VanderWeele, T.J., & Vansteelandt, S. Conceptual issues concerning mediation, interventions and composition. Statistics and Its Interface. 2009; 2, 457-468.

Valeri L, Vanderweele TJ. Mediation analysis allowing for exposure-mediator interactions and causal interpretation: theoretical assumptions and implementation with SAS and SPSS macros. Psychol Methods. 2013 Jun;18(2):137-50. doi: 10.1037/a0031034. Epub 2013 Feb 4. Erratum in: Psychol Methods. 2013 Dec;18(4):474. PMID: 23379553; PMCID: PMC3659198.

Azagba, S., Ebling, T., & Korkmaz, A. Social media and e-cigarette use: The mediating role of mental health conditions. Journal of affective disorders. 2024; 344, 528–534. https://doi.org/10.1016/j.jad.2023.10.053

5. It is unclear whether survey features were utilized in all models.

Response: Thank you for this comment. Yes, survey design features were incorporated in all analyses. Specifically, sampling weights were applied in all models to account for the complex survey design and to ensure nationally representative estimates. We have clarified this in the Methods section of the revised manuscript.

6. Stratify Table 1 by the exposure and report the standardized mean difference (SMD).

Response: We thank you for this crucial suggestion. Table 1 has been revised to present sample characteristics stratified by BMI category (underweight, normal weight, overweight, and obese). Standardized mean differences (SMD) were calculated using Cohen's w (√χ²/N) to quantify the magnitude of differences across BMI groups. The statistical analysis section has been updated to clarify the implementation of survey design features, and Table 1 has been revised accordingly in the manuscript.

7. Please explain why different reference categories were used in Table 2.

Response: Thank you for this comment. The use of different reference categories was intentional to facilitate interpretation of the association across the full BMI spectrum. Model 1 used underweight as the reference category to assess how higher BMI categories differ relative to the lowest BMI group. Model 2 used obese as the reference category to provide complementary comparisons from the opposite end of the BMI distribution. This approach allows for more complete pairwise comparisons across BMI categories without changing the underlying model structure and is consistent with recommendations that the choice of BMI reference category should be explicitly justified and that different reference categories can yield complementary insights (Flegal et al., 2014). We have clarified this in the Table 2 notes and Methods section while citing the rationale.

References:

Flegal, K. M., Kit, B. K., & Graubard, B. I. Body mass index categories in observational studies of weight and risk of death. American journal of epidemiology. 2014; 180(3), 288–296. https://doi.org/10.1093/aje/kwu111

Pavela, G., Yi, N., Mestre, L., Lartey, S., Xun, P., & Allison, D. B. The associations between relative and absolute body mass index with mortality rate based on predictions from stigma theory. SSM - Population health. 2022; 19, 101200. https://doi.org/10.1016/j.ssmph.2022.101200

8. This study’s cross-sectional nature prevents evaluating the temporal relationship between the exposure, mediator and outcome. That should be thoroughly discussed.

Response: We appreciate this important point. We agree that the cross-sectional nature of the data is a significant limitation that requires thorough discussion. We have expanded the limitations section to explicitly address the inability to establish temporal ordering between BMI, depression medication use, and smoking status, and have discussed the implications of this limitation for the interpretation of the mediation pathways.

Reviewer #1:

1. Abstract – Results Clarity

The results reported in the abstract are unclear and lack sufficient quantitative detail. The authors should clearly state the main statistical findings (e.g., direction and magnitude of associations) to improve transparency and consistency with the Results section.

Response: Thank you for your comments. We have revised the abstract section by adding some details about the direction and magnitude of association of the main statistical findings within the text limit for the abstract section.

2. Abstract – Structure and Focus

The abstract requires substantial revision to improve coherence. The objectives, methods, results, and conclusions should be more clearly delineated, and redundancy should be minimized.

Response: Thank you for your comments. We have revised the text of the abstract section to highlight the objectives, methods and results and conclusion in a clearer manner.

3. Keywords

Generalized Structural Equation Model should not be included as a keyword. Keywords should reflect the main scientific concepts rather than analytical techniques.

Responses: Thank you for your comments. We have deleted Generalized Structural Equation Model from our keyword list as advised.

4. Abbreviations and Terminology

All abbreviations (e.g., NCDs) must be defined at first mention and used consistently throughout the manuscript. Inconsistent terminology reduces readability and may confuse readers.

Response: Thank you for your comments. We have edited the text to so that acronyms are defined on first mention. We have also worked on consistency with the use of terms throughout the text to improve its readability.

5. Introduction – Rationale and Novelty

The introduction would benefit from a clearer statement of the study’s novelty. The authors should better articulate how this study adds to existing literature and what specific knowledge gap it addresses.

Response: Thank you for your comments. Our study is unique because it assesses the effect of a moderator (i.e, use of depression medication) on cigarette smoking across various Basal Metabolic Index (BMI) categories classified in our study (underweight, healthy weight, overweight and obese. Previous studies have mainly focused on whether participants were obese or not whereas our study provides insight into the relationship between depression medication use and cigarette smoking across all the BMI categories. An understanding of this role is important in planning cigarette smoking cessation interventions in health care settings where clients may be experiencing depression.

6. Methods – Variable Definition

The definition and operationalization of key variables, particularly depression medication use, should be clarified. The authors should justify the use of medication as a proxy and discuss potential misclassification.

Responses: We used depression medication as a proxy for diagnosis of depression among respondents because of the unavailability of data on depression on the NHIS dataset. The potential for misclassification has been highlighted in the limitations section in which we note that some individuals may have depression without being prescribed depression medication while also excluding people receiving non-pharmacological interventions to treat their depression.

7. Methods – Statistical Analysis

The statistical analysis section requires clearer explanation, particularly regarding model specification, reference groups, and interpretation of direct and indirect associations. This will enhance reproducibility.

Response: Thank you for your comments. We have revised the statistical analysis text of the Materials and Methods section to highlight the specification of our models, the reference groups used, and interpretation of direct and indirect associations.

8. Results – Presentation and Consistency

The results section needs further refinement. Some findings are difficult to follow, and clearer linkage between text, tables, and figures is required. Redundant reporting should be reduced.

Response: Thank you for your comments. We have edited the text of the results section linking the text to the tables. We have also eliminated redundant phrases in reporting our results.

9. Tables and Figures

Tables and figures should be carefully checked for clarity, labeling, and consistency with the text. All abbreviations used in tables should be defined in footnotes.

Response: Thank you for your comments. We have redrawn Tables 1 and 2 to ensure greater clarity of the information and reduced labelling errors. We have also labelled each table with all acronyms defined either in the body of the table or in the footnotes.

10. Discussion – Interpretation of Findings

The discussion should more critically interpret the findings rather than restating results. Alternative explanations and comparison with prior studies should be expanded.

Response: Thank you for your comments. We have edited the Discussion section to better compare our findings with that obtained in other studies and provided explanations to why some of our results either agree or contrast with that which was obtained elsewhere.

11. Conclusions and Limitations

The Conclusions and Limitations sections appear partially duplicated. These sections should be clearly separated, with conclusions summarizing implications and limitations addressing methodological constraints.

Response: Thank you for your observation. We have relabeled the Conclusions section correctly and edited the Limitations section highlighting the methodological constraints of our study and suggesting how future studies can improve on our methodology.

12. Language and Grammar

The manuscript contains several grammatical errors and typographical issues. A thorough language edit is recommended to improve clarity and professionalism.

Response: Thank you for your comments. We have taken time to edit the text to correct grammatical errors and limit typographical issues.

13. Ethical and Reporting Standards

Ethical statements and data descriptions should be reviewed to ensure full compliance with journal guidelines and reporting standards.

Response: Thank you for your comments. We have reviewed our ethical statement and it appears to be in line with the requirements of the PLOS One journal.

Attachment Submitted filename: Response to Reviewers.docx

# Editor report: Decision Letter 2

**DOI:** 10.1371/journal.pone.0351210.r005

**Authors:** Belal Hossain

26 May 2026

Mediating role of depression medication on association between Body Mass Index and cigarette smoking among US adults: Insights from the NHIS

PONE-D-25-35034R2

Dear Dr. Onigbogi,

We’re pleased to inform you that your manuscript has been judged scientifically suitable for publication and will be formally accepted for publication once it meets all outstanding technical requirements.

Within one week, you’ll receive an e-mail detailing the required amendments. When these have been addressed, you’ll receive a formal acceptance letter and your manuscript will be scheduled for publication.

An invoice will be generated when your article is formally accepted. Please note, if your institution has a publishing partnership with PLOS and your article meets the relevant criteria, all or part of your publication costs will be covered. Please make sure your user information is up-to-date by logging into Editorial Manager at Editorial Manager® and clicking the ‘Update My Information' link at the top of the page. For questions related to billing, please contact billing support.

If your institution or institutions have a press office, please notify them about your upcoming paper to help maximize its impact. If they’ll be preparing press materials, please inform our press team as soon as possible -- no later than 48 hours after receiving the formal acceptance. Your manuscript will remain under strict press embargo until 2 pm Eastern Time on the date of publication. For more information, please contact onepress@plos.org.

Kind regards,

Belal Hossain, PhD

Academic Editor

PLOS One

Additional Editor Comments (optional):

Reviewers' comments:

# Editor report: Acceptance letter

**DOI:** 10.1371/journal.pone.0351210.r006

**Authors:** Belal Hossain

PONE-D-25-35034R2

PLOS One

Dear Dr. Onigbogi,

I'm pleased to inform you that your manuscript has been deemed suitable for publication in PLOS One. Congratulations! Your manuscript is now being handed over to our production team.

At this stage, our production department will prepare your paper for publication. This includes ensuring the following:

* All references, tables, and figures are properly cited

* All relevant supporting information is included in the manuscript submission,

* There are no issues that prevent the paper from being properly typeset

You will receive further instructions from the production team, including instructions on how to review your proof when it is ready. Please keep in mind that we are working through a large volume of accepted articles, so please give us a few days to review your paper and let you know the next and final steps.

Lastly, if your institution or institutions have a press office, please let them know about your upcoming paper now to help maximize its impact. If they'll be preparing press materials, please inform our press team within the next 48 hours. Your manuscript will remain under strict press embargo until 2 pm Eastern Time on the date of publication. For more information, please contact onepress@plos.org.

You will receive an invoice from PLOS for your publication fee after your manuscript has reached the completed accept phase. If you receive an email requesting payment before acceptance or for any other service, this may be a phishing scheme. Learn how to identify phishing emails and protect your accounts at https://explore.plos.org/phishing.

If we can help with anything else, please email us at customercare@plos.org.

Thank you for submitting your work to PLOS ONE and supporting open access.

Kind regards,

PLOS ONE Editorial Office Staff

on behalf of

Dr. Belal Hossain

Academic Editor

PLOS One
